# Flexible Sensors for Battery Health Monitoring

**DOI:** 10.1007/s40820-025-01999-4

**Published:** 2026-01-05

**Authors:** Xin Wang, Haiyan Zhang, Xinyi Qi, Sheng Chen, Zekai Huang, Jinwei Zhao, Yihang Wang, Dezhi Wu, Gaofeng Zheng, Chenyang Xue, Jianlin Zhou, Hailong Wang, Zongyou Yin, Libo Gao

**Affiliations:** 1 Pen-Tung Sah Institute of Micro-Nano Science and Technology, Xiamen University, Xiamen, 361102 People’s Republic of China; 2 Shenzhen Research Institute of Xiamen University, Xiamen University, Shenzhen, 518000 People’s Republic of China; 3 Science and Technology On Vacuum Technology and Physics Laboratory, Lanzhou Institute of Physics, Chinese Academy of Space Technology, Lanzhou, 730000 People’s Republic of China; 4 School of Electronic Science and Engineering (National Model Microelectronics College), Xiamen University, Xiamen, 361102 People’s Republic of China; 5 Shenzhen Modulus Technology Co., Shenzhen, 518054 People’s Republic of China; 6 Innovation Laboratory for Sciences and Technologies of Energy Materials of Fujian Province (IKKEM), Xiamen, 361102 People’s Republic of China; 7 Research School of Chemistry, Australian National University, Canberra, ACT 2601 Australia

**Keywords:** Lithium battery, Battery health monitoring, Flexible sensing technology, Safety, Artificial intelligence

## Abstract

With the widespread application of lithium batteries in electric vehicles and energy storage systems, battery-related safety and reliability issues have become increasingly prominent. Conventional monitoring methods often struggle to address dynamic changes under complex operando. In recent years, flexible sensing technology has emerged as a promising solution for battery health monitoring due to its high adaptability and conformability to complex structures. Meanwhile, empowered by artificial intelligence (AI) for data analysis, the collected data enables efficient and accurate state assessment, offering robust support for accident prevention. Against this background, this paper first explores the integrated applications of flexible sensors in battery health monitoring and their unique advantages in addressing complex battery operating conditions, while analyzing the potential of AI in battery state analysis. Subsequently, it systematically reviews mainstream flexible sensing technologies (e.g., film sensors, thermocouples, and optical fiber sensors), elucidating their mechanisms for revealing intricate internal battery processes during operation. Finally, the paper discusses AI’s role in enhancing monitoring efficiency and accuracy, and envisions future research directions and application prospects. This work aims to provide technical references for the battery health monitoring field as well as promote the application of flexible sensing technologies in improving battery system safety and reliability.

## Highlights

- Flexible sensing technology enables battery health monitoring under complex operating conditions, overcoming the limitations of traditional monitoring methods.
- Artificial intelligence (AI) -powered data processing facilitates the construction of a "sensing–AI–control" framework, enhancing monitoring efficiency.

## Graphical abstract

**Figure:**
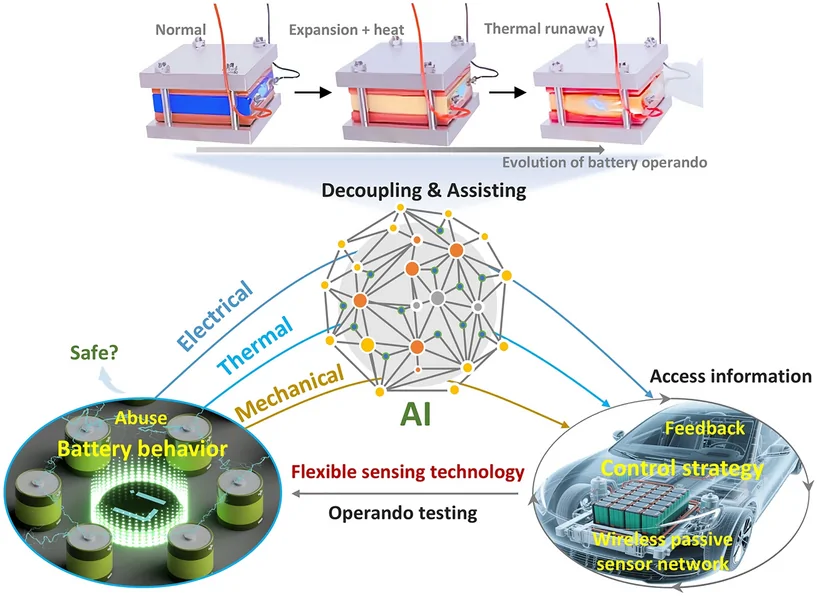

## Introduction

**Fig. 1:**
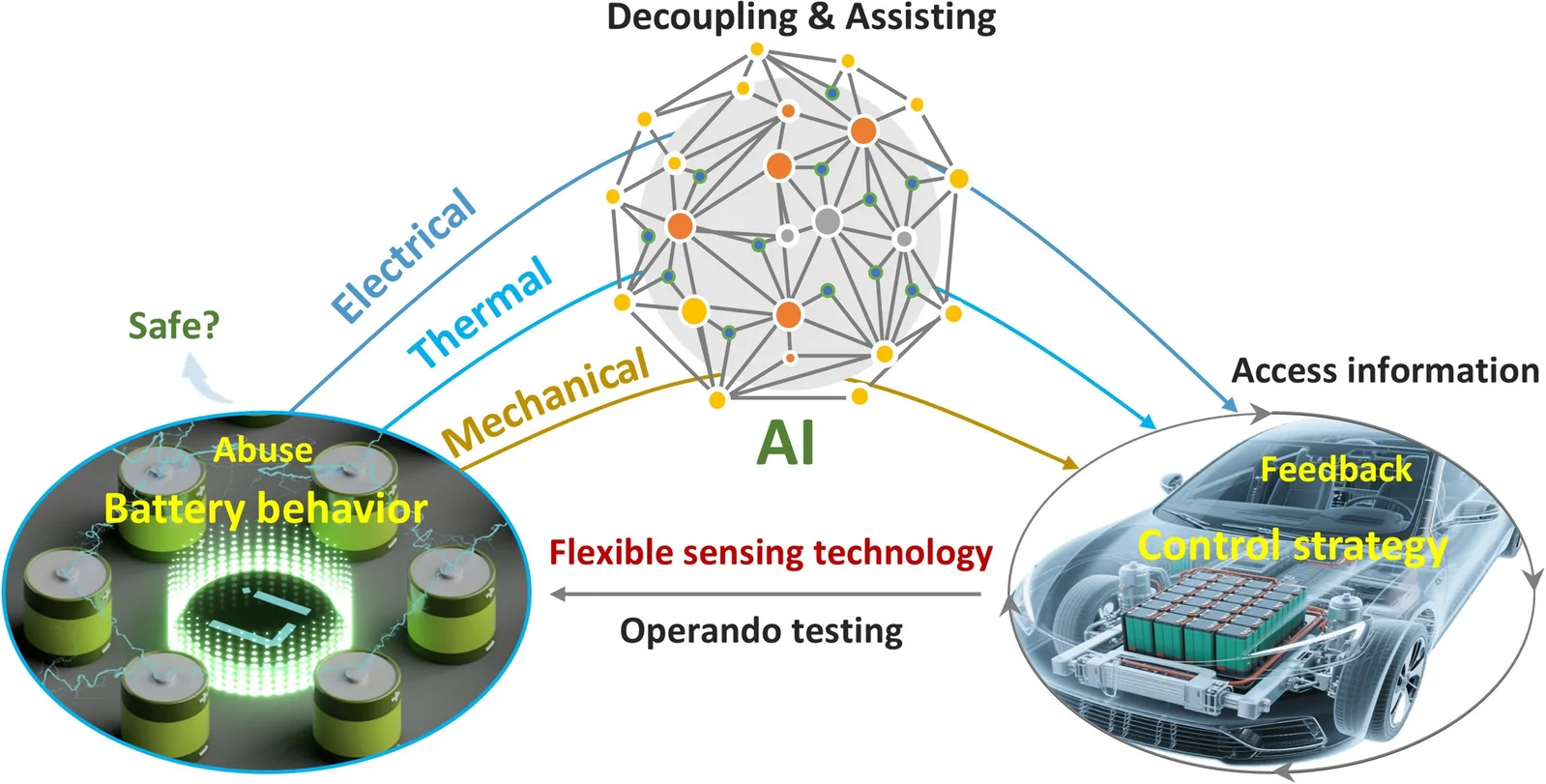
Battery health monitoring platform under operando

Lithium-ion batteries are the core components of electric vehicles and scaled energy storage systems [1–3]. The safety and health of these batteries directly determine the reliability and lifetime of energy systems [4–7]. Despite the substantial enhancement in energy density and cycling performance of batteries in recent years [8–11], under complex operando (e.g., mechanical abuse, thermal abuse, or electrical abuse), the coupling failure of multiple physical fields (mechanical, thermal, and electrochemical) within the battery may still trigger catastrophic events such as cascading thermal runaway or even explosion [12–15], resulting in serious safety hazards. The demand for battery testing has led to significant advancements in non-in situ and in situ techniques over the past few decades [16–18], with these techniques becoming increasingly important in the design of batteries [19, 20]. Nevertheless, the parameters that can be extracted by non-in situ and in situ techniques are frequently detached from the real operating state, i.e., the working environment. Consequently, battery operando detection technology has become a hotspot and a challenge in battery research in recent years [21–25]. Flexible sensing technology provides a breakthrough solution to this challenge by virtue of its thinness (thickness can be as low as micron level) [26–30], high ductility, and low invasiveness. It is capable of real-time monitoring of multi-physical field states under complex battery operando [31–36], and provides real-time feedback to the control system through the parsing system (Fig. 1). The in-depth integration of AI technology enables the monitoring system to extract multi-physical field correlation features (e.g., pressure-internal resistance coupling) from the massive data of the battery operando and establish the complex relationship between these key features and the battery performance to enhance the prediction and synergistic capability of the system and synergistic capabilities [37–42]. For example, in a typical logic closed loop, the electrical, temperature, and pressure signals of the battery system operation acquired by the sensors are synchronously analyzed by the AI model in real time [43, 44]. When battery abnormalities occur, the AI model is capable of detecting it in a timely manner and making a prompt regulation judgment, thereby enhancing the safety and stability of the battery.

Presently, the global annual sales of electric vehicles exceed 17 million units (2024), signifying a substantial market presence [45]. However, the stringent requirements of UN38.3 certification for battery abuse testing [46], along with the urgent needs of BYD, Tesla Motors, Contemporary Amperex, and other companies for full life cycle management of batteries, have led to the limitations of traditional monitoring technologies (e.g., voltage/current sensors, rigid thermocouples) [47]. In highly integrated battery modules, which rely on thousands of discrete sensors, system complexity and cost remain high, and real-time tracking of cell-level stress distribution is not possible. In this context, flexible sensing technology has emerged as a pivotal breakthrough, with its capacity for high integration (a single sensor capable of monitoring pressure, temperature, and strain synchronously) and ultra-thin embeddable features (thickness < 200 μm). This technology has effectively addressed the “sensing blind spot” prevalent in large-scale battery packs. The incorporation of AI technology is poised to enhance the efficiency of detection information processing to a considerable extent. Here, this timely review summarizes the applications and prospects of flexible sensors and AI technologies for battery health monitoring.

**Fig. 2:**
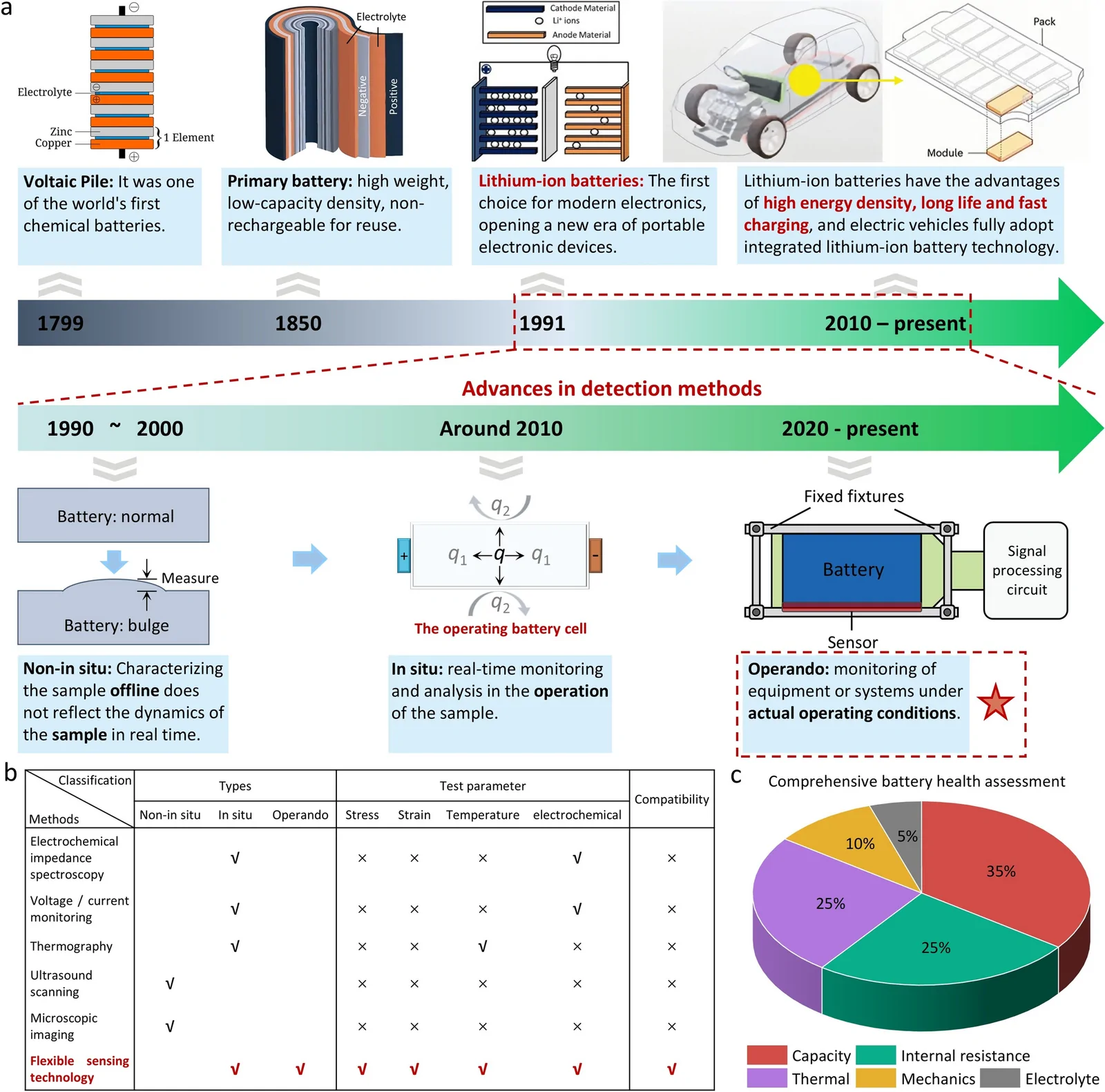
**a** Development history of batteries and their testing technologies; **b** Comparison of flexible sensing technology with other testing technologies; **c** Comprehensive assessment metrics for battery health status

The evolution of battery technology has consistently driven the innovation of testing methodologies. From the prototype of voltaic piles in 1799 to the practicalization of primary batteries in 1850 and subsequently to the commercial breakthrough of lithium-ion secondary batteries in 1991 [48–50], the increasing complexity of the battery systems has continuously propelled the development of testing technology in the direction of high precision and multidimensionality (Fig. 2a) [51–54]. This is particularly evident in the field of secondary batteries, as they need to undergo repeated charging and discharging cycles. The dynamic monitoring of their internal coupled multi-physics behaviors (e.g., electrode expansion triggered by lithium-ion migration, capacity degradation due to solid electrolyte interface (SEI) film thickening, temperature changes) poses significant challenges for assessing [55–57]. Early studies relied on non-in situ testing (e.g., post-disassembly dimensional measurements or offline electrochemical analyses). However, such methods study the battery pole piece under static conditions [58, 59], and the complex sampling process introduces many irrelevant factors, thereby hindering the capture of the transient response of the battery under real operando. With the development of operando testing techniques, researchers have been able to acquire fundamental parameters such as voltage and current during battery operation [60–63]. Flexible sensing technology, with its multiparameter compatibility (simultaneous acquisition of pressure, strain, temperature, and electrochemical signals), ultra-thin, scalable, and easy integrability capability, as well as the spatial resolution brought by arraying (Fig. 2b), is expected to be a key bridge between microscopic mechanisms and macroscopic characteristics. Based on the powerful capability of flexible sensing technology and the real state of the battery, a comprehensive and quantitative assessment of the battery’s state of health (SOH) can be realized, including capacity degradation, internal resistance change, temperature anomaly, mechanical deformation, and electrochemical parameters (Fig. 2c). These multidimensional indicators constitute key metrics for SOH evaluation [64].

Battery health monitoring has moved from offline analysis of a single parameter to the new stage of in situ multi-physical field sensing [65], and the breakthrough application of flexible sensing technology is reshaping the underlying logic of this field. By deeply integrating the sensor network with the battery body, researchers have been able to analyze the dynamic coupling mechanism of mechanical–thermal–electrochemical in both temporal and spatial dimensions, which not only provides a brand-new perspective for understanding the battery failure but also gives rise to the technological innovation of the “energy sensing” paradigm. In this paper, we systematically review the recent progress of flexible sensing technology in battery health monitoring [66]. First, this paper focuses on the way flexible sensors are combined with battery operando exploring AI-driven multi-source data fusion to improve the efficiency and accuracy of battery health monitoring [67, 68]. The paper then summarizes the commonly used means and key technologies for battery health monitoring, including film, thermocouple [69], and fiber optic sensors [70], and the monitoring of core parameters such as current, voltage, charge/discharge rate, and temperature. Finally, the paper further explores the role of AI technologies in battery health monitoring efficiency and provides an outlook on the direction of energy sensing and technological challenges. By systematically summarizing the whole-chain technology framework of “sensing-data-analysis” for battery health status, this review aims to provide theoretical support for battery health monitoring and identify new research opportunities for the future development of battery technology.

## Research Objective

Although a few reviews have focused on flexible pressure sensing technologies for battery monitoring [71–74], these studies are constrained by three key limitations: (1) Technical isolation: Most reviews concentrate on a single sensor type (e.g., film or fiber optics), lacking systematic analysis of the synergistic effects of multimodal flexible sensing technologies; (2) Insufficient dynamic monitoring capabilities: Existing reviews predominantly address static parameters (e.g., maximum pressure thresholds) while neglecting real-time tracking mechanisms for dynamic processes such as battery expansion and thermal runaway; (3) Data-decision disconnection: Traditional reviews fail to delve into the closed-loop logic between sensing data and AI-driven state analysis, resulting in a fragmentation between sensing technologies and analytical methods. This review bridges these gaps by integrating multi-physics sensing networks with an AI-enabled dynamic decision-making framework. This review has two primary objectives: (1) Systematically elucidating how flexible sensing technologies overcome the spatiotemporal resolution limitations of traditional monitoring through noninvasive integration and multiparameter synchronous perception, addressing the coupling of multi-physical fields (mechano-thermo-electrochemical) in complex battery operating conditions; (2) Revealing the pivotal role of AI in feature extraction, state estimation, and closed-loop control of sensing data, thereby establishing an integrated perception–analysis–decision framework.

## Apparatus and Method for Monitoring Battery Operation

**Fig. 3:**
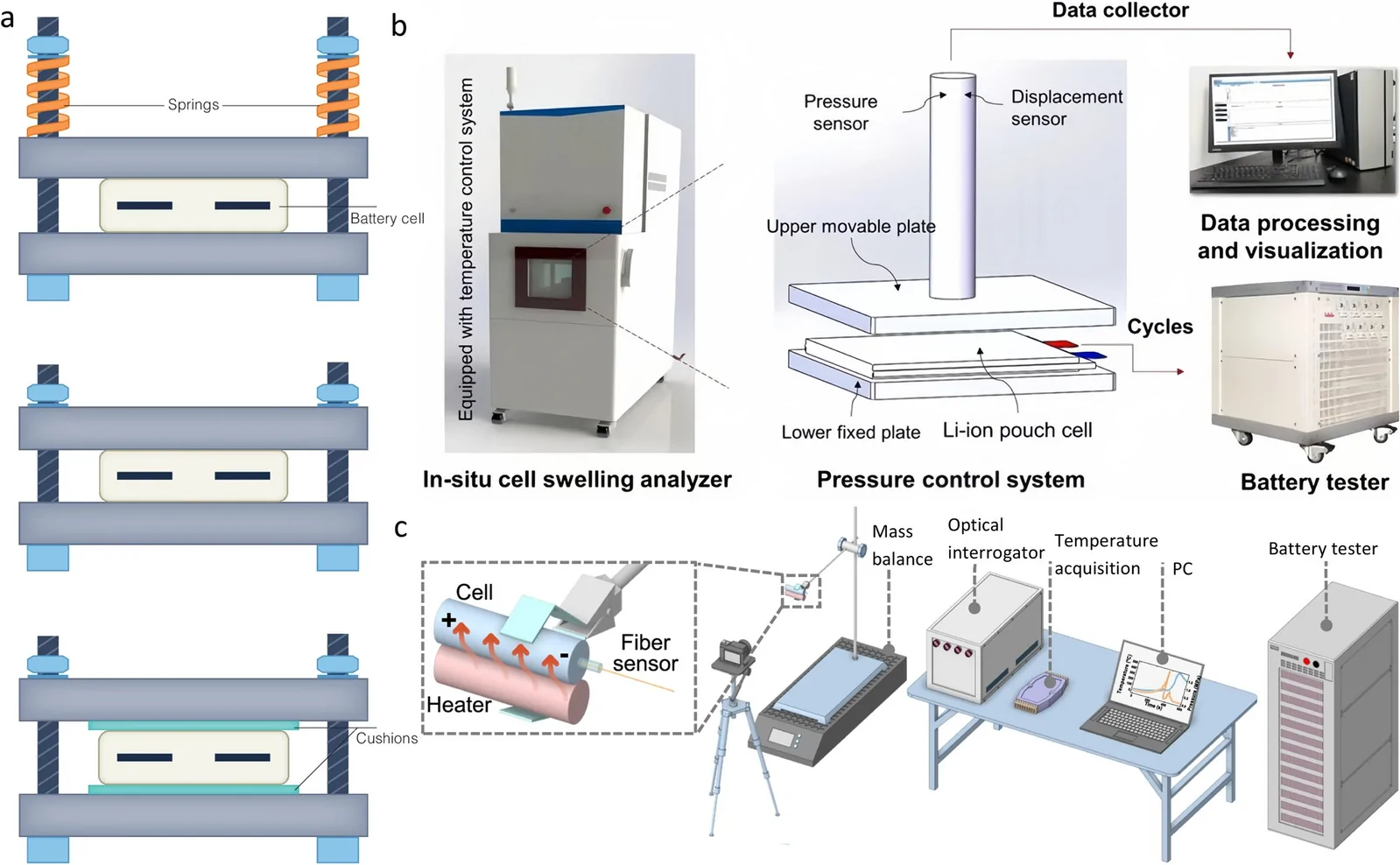
**a** Three main battery expansion force control devices. Copyright 2024, Springer Nature. Reproduced with permission [76]. **b** Test rig for prismatic and flexible pack batteries. Copyright 2023, American Chemical Society. Reproduced with permission [77]. **c** Test rig for cylindrical batteries. Copyright 2023, Springer Nature. Reproduced with permission [78]

In the characterization of the battery’s operando, the pressure control is very critical. At this stage, three main types of control are utilized, including a spring-controlled fixed pressure design [75], a screw-controlled fixed gap design, and a dual controlled pressure and gap design (Fig. 3a) [76]. The pressure distribution in the spring-controlled fixed pressure design depends on the stiffness of the spring and the form of the fixture plate. The disadvantage is poor pressure uniformity, especially during cycling and evolving pressure and expansion build-up phases. Fixed gap methods can provide uniform pressure distribution control; however, the pressure built up during cycling is ultimately uncontrollable, and the energy buildup predisposes the battery to damage and abnormal deformation. As illustrated in the bottom panel of Fig. 3a, the design of both pressure and gap is controlled by means of cushions and screws, which allows for the introduction of cushions of varying stiffness between the fixed plates on demand. This approach controls the pressure buildup inside the battery, providing a more reliable device for correlating battery performance with design parameters. The clamping strategy of the fixture is structured to precisely regulate the pressure dynamics inside the battery by means of mechanical confinement, which directly affects the micro-processes of the electrochemical reactions, making the monitoring signals reflect the intrinsic characteristics of the battery more realistically.

The structure of lithium batteries encompasses three primary categories: prismatic, pouch, and cylindrical. Each category exhibits distinct physical characteristics and is suitable for specific application scenarios. Prismatic batteries are widely used in various portable electronic devices due to their high energy density and commendable mechanical strength; pouch batteries have become the first choice for high-end smartphones and electric vehicles due to their thinness and high safety; cylindrical batteries occupy an important position in many application scenarios due to their mature production process and low cost. Stress measurement techniques adapted to the structural characteristics of different batteries also take different forms from each other. Prismatic and pouch batteries are suitable for parallel-plate fixtures (Fig. 3b) [77]. They can provide uniform pressure to ensure the accuracy of the test results and facilitate the installation of sensors on the surface or inside the battery for real-time monitoring of key performance indicators such as voltage, current, and temperature. As for cylindrical batteries, due to their uneven surface, they are usually clamped with mechanical clamps and combined with implantable fiber optic sensors to monitor the internal state of the batteries (Fig. 3c) [78]. The fiber optic sensors are capable of transmitting real-time information about the stress, strain, and temperature inside the battery [79], which provides powerful data support for the health management of the batteries.

**Fig. 4:**
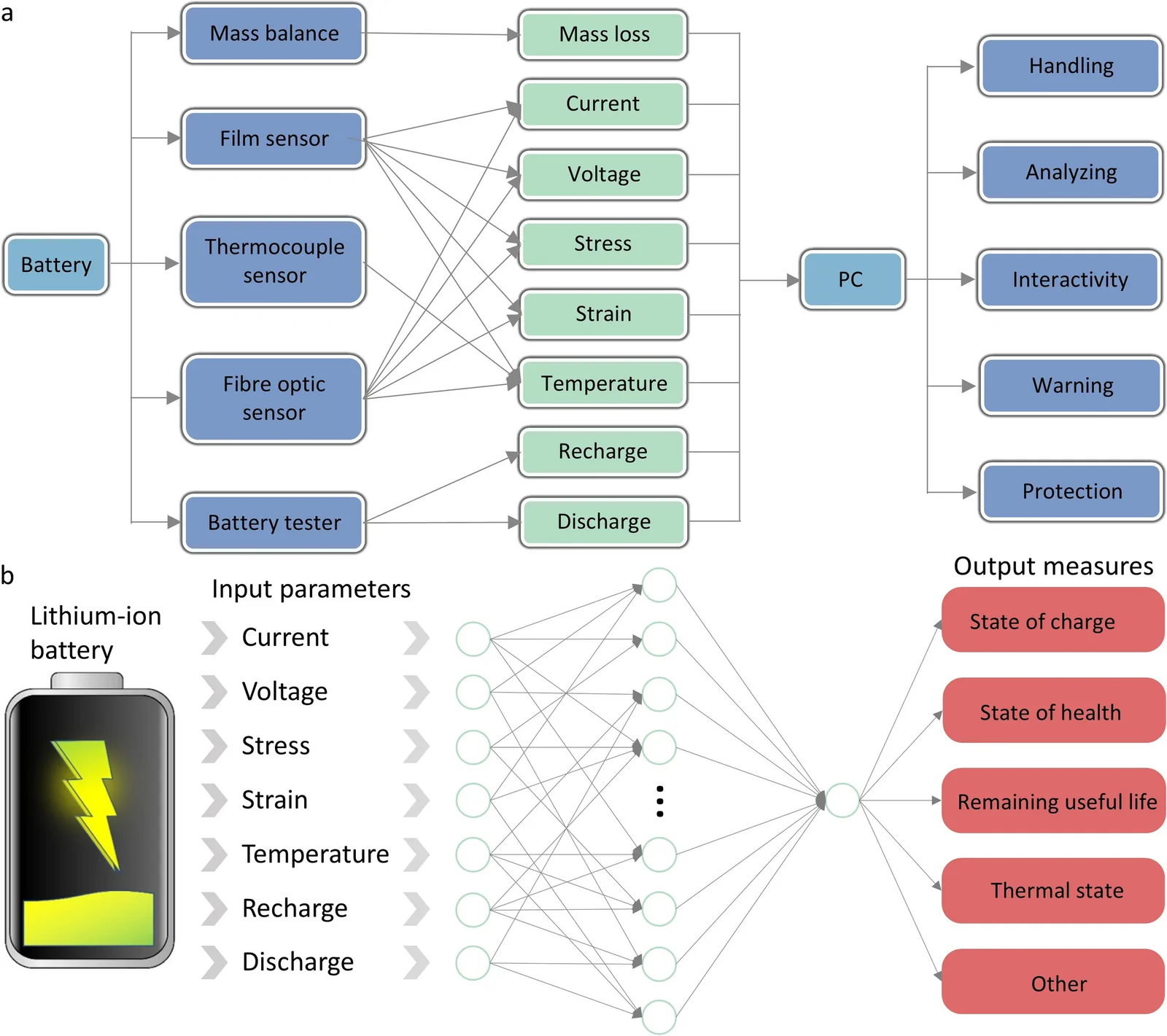
**a** Testing and characterization process of lithium batteries. Copyright 2023, Springer Nature. Reproduced with permission [78]. **b** Algorithmic analysis process of the lithium-ion battery monitoring system to achieve AI empowerment. Copyright 2024, Elsevier. Reproduced with permission [74]

Data collection, processing, and analysis are the key links to ensure stable battery performance and safe operation. Through the comprehensive use of a variety of high-precision instruments such as balances, sensors, and battery testers, key information such as mass loss, current change, voltage fluctuation, stress, strain, temperature difference, and charge/discharge rate of the battery can be comprehensively collected (Fig. 4a) [78]. These data not only reflect the current state of the battery but also reveal trends and potential problems in its long-term use. Multidimensional data are integrated through an advanced host computer system. Scientific algorithms are designed in the host computer to dig deep into the multidimensional data features to reflect the health status of the battery in real time. What’s more, when abnormalities are detected, the monitoring system is able to immediately trigger an early warning mechanism and automatically execute a series of protective measures, thus ensuring the safe and stable operation of the battery.

In battery health management, a real-time warning and protection mechanism is the core technology to ensure battery safety and prolong service life [80]. Through efficient data processing, the system is able to issue an early warning immediately when abnormal conditions are detected and trigger the execution of protective measures, such as adjusting charging strategies, limiting output power, or even powering down. These measures not only prevent dangerous situations such as overcharging, over-discharging, or overheating, but also optimize the efficiency of the battery and extend its service life. In addition, early warning and protection mechanisms include monitoring the environment in which the battery is used [81], such as temperature and humidity control, to ensure that the battery operates under optimal conditions. Through these comprehensive early warning and protection measures, Li-ion batteries are able to provide a stable and reliable energy supply in a variety of application scenarios, providing a solid guarantee for the normal operation of electronic equipment. Figure 4b illustrates the parsing and decision-making process of an AI-enhanced lithium-ion battery monitoring system [74]. The system analyzes and predicts various output measurements of the battery, including state of charge, state of health, remaining useful life, thermal state, and other relevant metrics by acquiring multiple input parameters (e.g., current, voltage, stress, strain, temperature, state of charge, and state of discharge). This system helps to monitor battery performance in real time, improving the safety and efficiency of the battery.

This chapter systematically reviews three mainstream battery expansion force control devices (spring-controlled, gap-controlled, and hybrid-controlled), highlighting the critical role of fixture design in characterizing intrinsic battery properties. By comparing sensing solutions adapted to prismatic, pouch, and cylindrical batteries (e.g., parallel-plate fixtures and fiber optic implantation), structural compatibility is identified as a prerequisite for precise monitoring. Furthermore, the synergistic framework of data acquisition and AI-driven analysis provides a methodological foundation for real-time correlation of multidimensional parameters (stress, temperature, SOC), signifying a paradigm shift in battery monitoring from offline single-parameter to online multi-physics approaches.

## Flexible Sensor Technology for Battery Health Monitoring

**Fig. 5:**
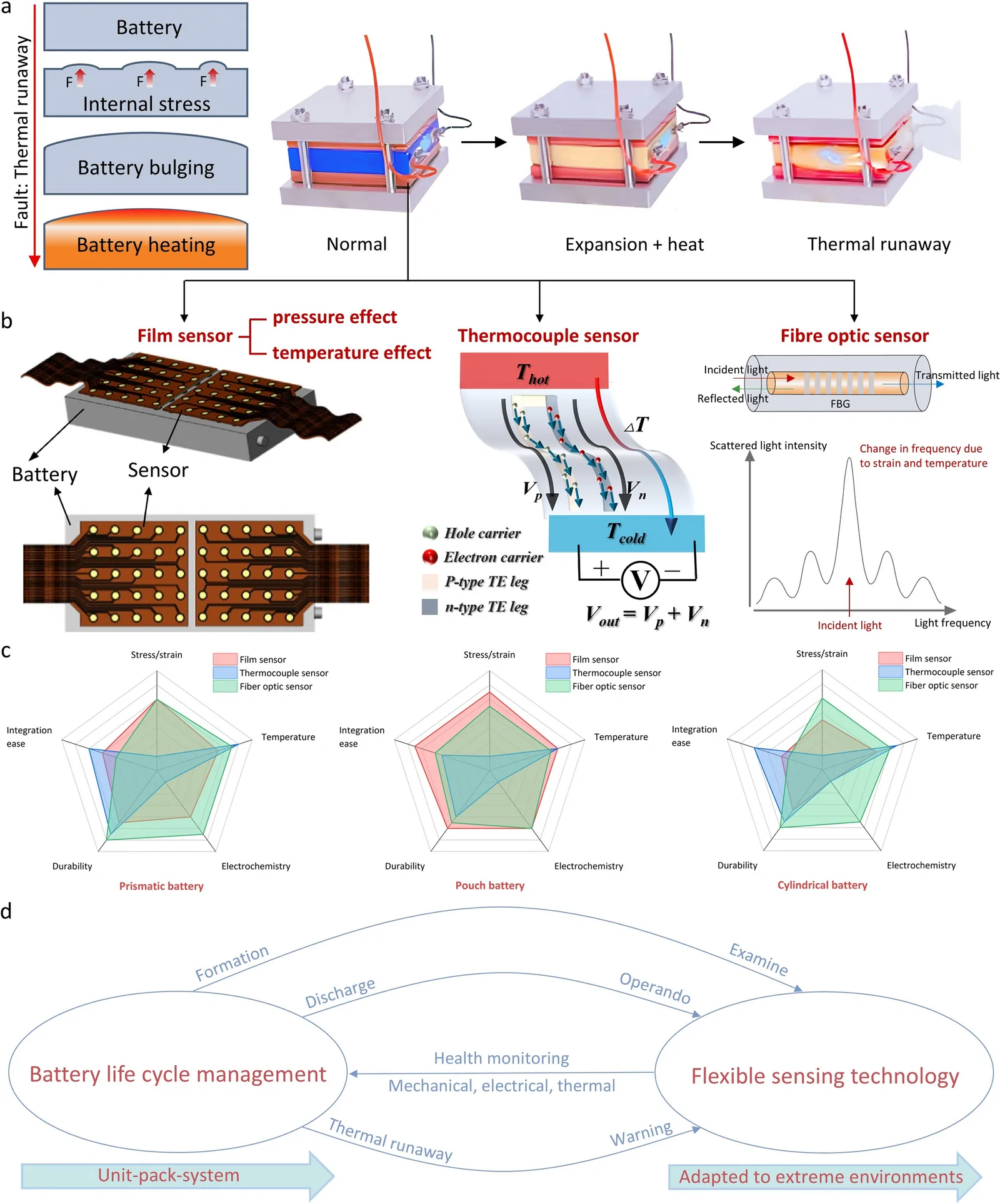
**a** The process of thermal runaway in Li-ion batteries and the conceptual diagram of the Li-ion battery testing device. Copyright 2024, Elsevier. Reproduced with permission [82]. **b** Three types of flexible sensor technologies involved in lithium battery safety monitoring. Copyright 2016, Elsevier. Reproduced with permission [83]. Copyright 2023, Elsevier. Reproduced with permission [84]. Copyright 2022, Springer Nature. Reproduced with permission [85]. **c** Adaptation study of different types of sensors. **d** Logic diagram of the application of flexible sensing technology in the full life cycle health monitoring of batteries

The combination of flexible sensor technology and lithium battery health monitoring marks a new stage in battery monitoring technology. Traditional rigid sensors are limited by physical form and monitoring dimension. Capturing the complex internal state changes during lithium battery charging and discharging remains challenging. Flexible sensors, with their ultra-thin, stretchable, and highly sensitive properties, can be seamlessly attached to battery surfaces or integrated into modules to enable real-time monitoring of battery expansion, local temperature rise, and stress distribution. This approach represents a new stage in accurate, dynamic, and intelligent battery health management. Changes in battery state, especially increases in internal pressure, bulging phenomena, thermal effects, and ultimately the potential for thermal runaway (Fig. 5a) [82], are issues that need to be prioritized in lithium battery health monitoring. The easy integration of flexible sensors allows them to be coupled to the battery system without changing the battery operando, providing real-time operating feedback on the battery state to help to and mitigate these safety risks. These sensors are not only capable of monitoring the physical parameters of the battery but also predicting the battery’s state of health and remaining useful life through data analysis.

This review focuses on the application of flexible sensor technology itself in the safety monitoring of lithium batteries, including film [83], thermocouple [84], and fiber optic sensors (Fig. 5b) [85]. In the future, with the further development of flexible sensor technology in terms of sensitivity, sensing array density, sensing data categories, and flexibility, combined with the powerful analytical capabilities of AI, the health monitoring of lithium batteries will become more intelligent and efficient [86]. This provides a more solid guarantee for the safe operation of lithium batteries and also opens up new possibilities for the future development of battery technology. The applicable scenarios for these sensors are shown in Table 1. Furthermore, we focus on the application of different sensors in different batteries to analyze the performance and suitability of the sensors (Fig. 5c) [87–95]. It can be found that thin-film sensors are better suited for prismatic/cylindrical batteries, thermocouple sensors have the best performance for temperature sensing, and fiber optic sensors are more like a “hexagonal warrior” with excellent performance in a wide range of sensors.

**Table 1:** Application scenarios for sensors

Sensor types	Monitoring parameters	Battery types	Battery stages	References
Film	stress	prismatic	use	[94]
	Temperature	prismatic	formation, use	[83]
	Stress, temperature	pouch	formation, use, thermal runaway	[96]
	Electrochemistry	pouch	formation, use	[97]
	Electrochemistry	pouch	formation, use	[98]
Thermocouple	Temperature	pouch	formation, use	[99]
	Temperature	prismatic	use	[100]
Fiber optic	Temperature	prismatic	formation, use	[101]
	Strain, temperature	pouch	formation, use	[102]
	Stress, temperature	cylindrical	formation, use, thermal runaway	[78]
	Strain, temperature	pouch	formation, use	[103]
	Strain	prismatic	formation, use	[104]
	Electrochemistry	pouch	formation, use	[105]
	Stress, temperature	cylindrical	formation, use	[106]

Explain: The battery stage is generally divided into three stages, including the “formation stage” used to activate the battery, the “use stage” in applications, and the “thermal runaway stage” in the event of a failure

These sensors need to fulfill the testing needs under different operando conditions. The diversity of sensor performance (e.g., stress/strain, temperature, response time, durability) and the suitability of the battery structure differ significantly, which directly results in different integration methods. This paper summarizes the performance of different sensors (film, thermocouple, fiber optic) and gives the recommended integration methods to provide technical references for researchers and engineers (Table 2).

**Table 2:** Technical characteristics of flexible sensors

Sensor types (integration methods)	Substrates	Ranges (Stress or strain or temperature)	Response time	Durability	Sensitivity or resolution	Errors (estimated)	References
Film (attachment)	Polydimethylsiloxane (PDMS)	0–250 kPa, 0–20%, 20–80 °C	/	> 7000 cycles	0.172 °C^−1^, 610.2 kPa^−1^	10%	[107]
	Thermoplastic urethane (TPU)	0–100 kPa	120 ms	> 5000 cycles	3.997 kPa^−1^, 4.7 Pa	4.6%	[108]
	Polyethylene terephthalate (PET)	0–50 kPa, − 20–90 °C	17 ms	> 5000 cycles	804.27 kPa^−1^, 31.74 Pa	10%	[109]
	Polyimide (PI)	0–50 kPa, − 10–250 °C	120 ms	> 5000 cycles	158.23 kPa^−1^	6.8%	[110]
	Polyvinyl alcohol (PVA)	0–80 kPa, 20–50 °C	30 ms	> 10,000 cycles	6.45 kPa^−1^, 5 Pa	/	[111]
	Poly(styrene-b-ethylene-b-butylene-b-styrene) (SEBs)	0–250 kPa, 0–350%	179 ms	> 4000 cycles	0.155 kPa^−1^, 16 Pa	/	[112]
	Poly (acrylamide) (PAM)	0–175%, 6–36 °C	187 ms	> 18,000 cycles	1.8, 1%	35%	[113]
Thermocouple (attachment)	PI	0–10 N, 10–160 °C	/	> 5000 cycles	76.5 μV °C^−1^	3%-11%	[114]
	Paper/PDMS/Si_3_N_4_	20–200 °C	9.8 ms	> 1000 cycles	52.67 μV °C^−1^, 0.8 °C	/	[115]
	PDMS	0–40 °C, 20–80 °C	/	> 1000 cycles	22.3 μV °C^−1^	0.35%	[116]
Fiber optic (attachment, implantable)	Few-mode fiber	0–600 με, 25.3–58 °C	Typically less than 10 ms	0.5 h	-0.013 nm μϵ^−1^, 0.262 nm °C^−1^		[117]
	Fabry–Perot interferometer (FPI)	0.2–2 MPa, 30–200 °C		/	3.63 nm MPa^−1^, 9.22 pm °C^−1^	1.4%	[118]
	Thin-core fiber	0–100 kPa, 20–85 °C		1.5 h/	− 14.3 nm MPa^−1^, − 340 pm °C^−1^	< 0.1%	[119]
	Fiber Bragg grating, FPI	0–2 MPa, 25–600 °C		> 100 cycles	4.19 nm MPa^−1^, 10.3 pm °C^−1^	0.5%	[78]

In practice, the application of flexible sensors in the whole life cycle management of batteries is gradually deepening (Fig. 5d). In the “activation” phase of battery formation, flexible sensing technology has realized breakthrough applications. Through the high-precision sensor array integrated into the shell, the system can capture the micron-level deformation and temperature fluctuation of the battery cell during charging and discharging in real time, and work with the big data analytics platform to implement monitoring. This in situ monitoring technology is reshaping the standards of lithium-ion battery production. In the use phase, the sensor carries out long-term multidimensional sensing, a topic that has been widely discussed in academia and industry [120]. Expansion toward battery packs is being actively pursued with a view to realizing true health monitoring of batteries in their operando state, which in turn can be applied to large-scale battery packs and electric vehicles. When confronted with safety challenges, the most pressing issue is the real-time warning of thermal runaway [96], necessitating millisecond-level response from the sensor to facilitate timely emergency disposal. The incorporation of AI technology in this process is expected to enhance the operational efficiency of the system. It is important to note that flexible sensors must be adapted to complex and extreme working environments, including vibration, strong noise, extreme cold (− 40 °C), high temperature (200 °C), high altitude, and high humidity areas. Flexible sensing technology is rapidly evolving along the path of the “unit-pack-system.”

### Mechanisms of Sensors

**Fig. 6:**
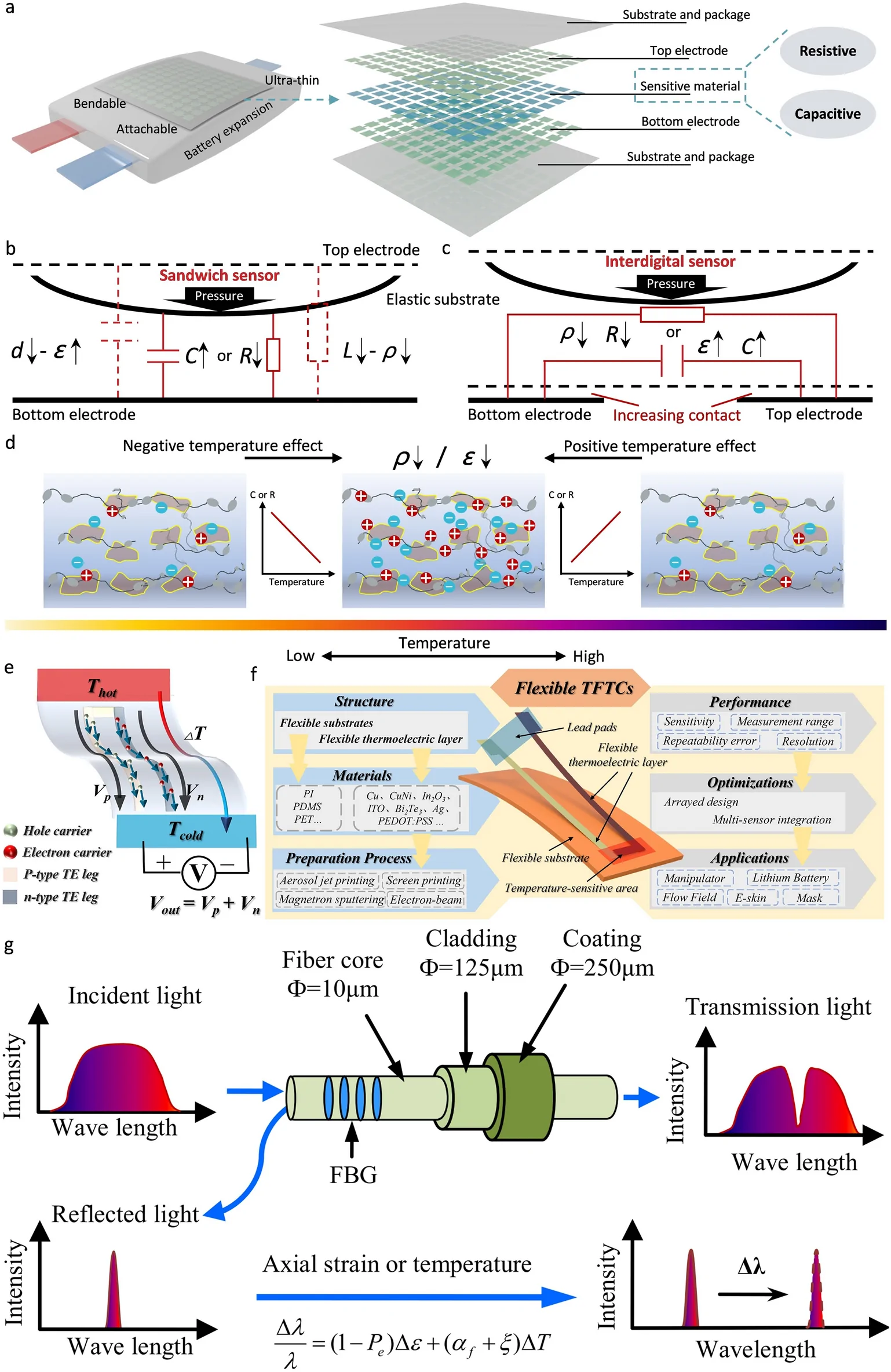
**a** Technical characteristics of film sensor. Sensing mechanisms of film pressure sensors: **b** Sandwich type and **c** Fork-finger type. **d** Sensing mechanism of the film temperature sensor. **e** Temperature measurement principle of film thermocouple sensors. Copyright 2023, Elsevier. Reproduced with permission [84]. **f** Multi-scenario application of TFTCs. Copyright 2023, Elsevier. Reproduced with permission [84]. **g** Principle of operation and sensing mechanism of fiber Bragg grating. Copyright 2018, Springer Nature. Reproduced with permission [128]

The sensor types covered in this review primarily include film pressure, film temperature, thermocouples, and fiber optic sensors. Film pressure sensor detection technology has important applications in battery stress detection [95]. This technique utilizes the high sensitivity and fast response characteristics of film sensors to accurately monitor the stress changes that occur in batteries during use. Figure 6a shows the technical characteristics of a thin-film pressure sensor for battery health monitoring. The sensor is ultra-thin and bendable, allowing it to perfectly adhere to the battery surface. The three most central features of conventional thin-film pressure sensors are the substrate and encapsulation layer, the electrode layer, and the sensitive layer, which together determine the mechanical–electrical characteristics of the sensor. The sensitive layer is subdivided into resistive and capacitive, which is determined by the conductive mechanism of the sensor. Two common film pressure sensor configurations are illustrated in Fig. 6b, c: sandwich sensors and fork-finger sensors [121]. In a sandwich-type sensor (Fig. 6b), pressure acts on the elastic substrate, reducing the distance between upper and lower electrodes, leading to an increase in dielectric constant or a decrease in resistivity, which in turn causes an increase in capacitance or a decrease in resistance. Fork-finger-type sensors (Fig. 6c), on the other hand, change the sensed signal by increasing the contact area. When pressure is applied to the sensor, the contact area between the bottom and top electrodes increases, causing an increase in dielectric constant or a decrease in resistivity, which further results in an increase in capacitance or a decrease in resistance. These two designs enable the sensor to effectively sense pressure changes and convert them into electrical signals. It is worth noting that both sandwich-type and fork-finger-type, under pressure, will lead to an increase in dielectric constant or a decrease in resistivity. This is due to the fact that when the dielectric or sensitive layer is pressurized, the internal structure is squeezed, the molecular density per unit volume is significantly increased, and the effective dielectric constant is increased, or more conductive pathways are formed, leading to an increase in dielectric constant or a decrease in resistivity. This characteristic is more evident in some sensors with microstructures. Film pressure sensors can provide real-time, accurate data in battery stress detection, which can help optimize battery design and improve battery performance and safety. By monitoring the stress distribution of batteries under different operando, potential safety hazards can be detected in a timely manner to prevent battery failure and damage [122].

Film temperature sensors show great capability in accurate temperature monitoring. Temperature monitoring is likewise a critical parameter for battery operando [103, 123, 124]. Changes in battery temperature not only affect the performance and lifespan of the device but are also directly related to the safety and reliability of the system. Figure 6d shows the sensing mechanism of the film temperature sensor, specifically the negative temperature coefficient (NTC) and positive temperature coefficient (PTC), which is determined by the temperature–electrical effect of the temperature–sensitive material [125]. In the NTC, as the temperature increases, the electronic or ionic activity inside the sensor increases, and the resistivity or dielectric constant of the sensor decreases, which in turn leads to a decrease in the resistance (R) or capacitance (C) of the sensor. Conversely, in the PTC, an increase in temperature leads to an increase in resistance or capacitance. This effect is usually due to thermal expansion or a thermally activated process in the material. This unique temperature response mechanism of film temperature sensors enables them to provide highly accurate temperature monitoring over a wide range of temperatures. Through precise control of the material and structure of the sensor, a sensitive response to temperature changes can be achieved to meet a variety of battery temperature monitoring needs [96].

Thermocouple sensors are another type of sensor used for temperature measurement, operating on the principle of the Seebeck effect [126], which describes the electric potential generated in a circuit due to a temperature difference when two dissimilar metals are in contact. When two conductors of different materials form a closed circuit and there is a temperature difference between their ends, thermal energy is converted into electrical energy, which generates an electric current in the circuit. Specifically, a thermocouple consists of two conductors of different materials, and when there is a temperature gradient between their hot (*T*_hot_) and cold (*T*_cold_) ends, the charge carriers (e.g., electrons and holes) within the conductors move in a specific direction. This movement results in a potential difference (*V*_out_) at the ends of the thermocouple that is proportional to the temperature difference. Because of the metal electrode contact, it is equivalent to a film temperature sensor that is more stable and can adapt to a more severe test environment. Figure 6e illustrates the principle of temperature measurement in a flexible thermocouple (TFTC) [84]. In this figure, the hot end (*T*_hot_) is in contact with the object to be measured, while the cold end (*T*_cold_) is connected to a measurement system. By measuring the potential difference (*V*_out_) between the cold end and the hot end, the temperature of the hot end can be deduced, which in turn tells the temperature of the measured object. Thermocouples utilize the Seebeck effect to indirectly measure temperature by measuring the potential difference caused by the temperature difference. This method is widely used in a variety of temperature measurement scenarios due to its simplicity, reliability, and independence from an external power supply.

Film thermocouple sensors (TFTCs) are advanced sensors with flexible substrates and thermoelectric layers that provide highly accurate temperature monitoring in a wide range of application scenarios (Fig. 6f) [84]. The structure consists of a flexible substrate and a flexible thermoelectric layer using a variety of materials such as substrate materials (PI, PDMS, PET, etc.) and thermoelectric layer materials (Cu, CuNi, In_2_O_5_, ITO, Bi_2_Te_3_, Ag, PEDOT/PSS, etc.). The preparation process involves techniques such as aerosol jet printing, screen printing, magnetron sputtering, and electron beam evaporation. In terms of performance, the sensors require high sensitivity, wide measurement range, low repeatability error, and high resolution. The performance is further enhanced by optimization measures such as array design and multi-sensor integration. The application areas are widely applied, including manipulators, lithium batteries, flow fields, electronic skins, and masks. Overall, TFTCs demonstrate significant advantages in terms of structural design, material selection, preparation process, performance optimization, and application areas, making them one of the excellent choices for battery temperature health monitoring.

Fiber optic sensors offer significant advantages in battery health monitoring due to their high sensitivity, versatility, and ease of integration [127]. They can penetrate deeply into the battery and monitor key parameters such as temperature, strain, stress, pressure, and charge/discharge rate in real time, providing comprehensive data on battery conditions to prevent thermal runaway, optimize performance, and improve design. Figure 6g illustrates the working principle and sensing mechanism of a fiber Bragg grating (FBG) [128]. This fiber grating is fabricated by periodically modulating the refractive index within the fiber core. As broadband light propagates through the fiber core, the grating reflects a narrowband portion of the broadband light in a specific wavelength range and lets the rest of the broadband light pass through. Once the incident light enters the fiber core and reaches the grating, the presence of the fiber grating causes the wavelengths of the reflected and transmitted light to shift when axial strain or temperature changes occur. Finally, the wavelength and intensity distributions of the incident, reflected, and transmitted light are decoupled to reverse the strain or temperature changes.

### Battery Expansion Force Detection

**Fig. 7:**
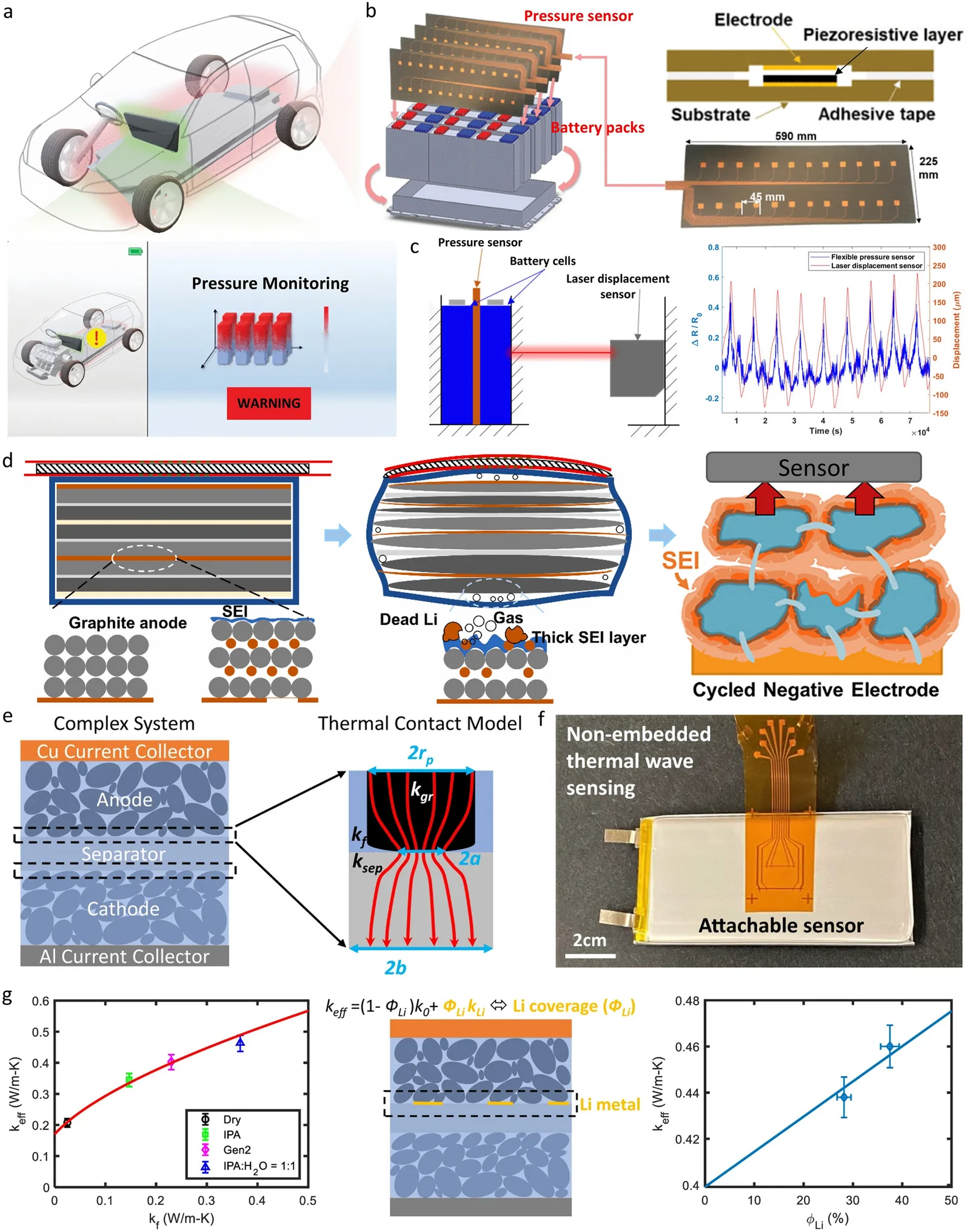
**a** Schematic of a battery expansion monitoring system integrated in an electric vehicle. Copyright 2024, IEEE. Reproduced with permission [94]. **b** Application of film sensors for on-board battery expansion force detection. Copyright 2024, IEEE. Reproduced with permission [94]. **c** Pressure calibration and characterization of on-board batteries. Copyright 2024, IEEE. Reproduced with permission [94]. **d** Healthy and swollen phase layers of the solid electrolyte interface of the battery in operando. Copyright 2019, Elsevier. Reproduced with permission [133]. **e** Internal structure of a commercial battery. Copyright 2023, Springer Nature. Reproduced with permission [97]. **f** Integration method of the non-embedded thermal wave sensor. Copyright 2023, Springer Nature. Reproduced with permission [97]. **g** Relationship between the thermal conductivity of the battery and the thermal conductivity of the electrolyte and lithium coverage. Copyright 2023, Springer Nature. Reproduced with permission [97]

Film pressure sensor detection technology demonstrates a number of advantages in the detection of mechanical pressure on battery surfaces [129, 130]. It has high sensitivity, good linearity, and accurate pressure measurement capability, and it can carefully capture the small pressure changes on the battery surface. Its thin and flexible structure can adapt to the different shapes and contours of the battery surface and fit tightly to ensure the accuracy of the measurement. The technology is highly stable and can work reliably under different environmental conditions [131, 132]. Recently, Lei Shao et al., based on the industrialized application scenario of new energy vehicles (Fig. 7a) [94], proposed a flexible film pressure sensor based on flexible printed circuit board (FPCB), which can be integrated between battery packs (Fig. 7b) for continuous monitoring of battery expansion to address the potential danger of thermal runaway of the batteries. In the electric vehicle battery expansion monitoring system, the battery pack is situated at the bottom of the vehicle and has flexible pressure sensors installed inside, which are positioned between the battery cells to monitor the expansion of the battery during charging and discharging. The FPCB is fabricated based on the high-temperature-resistant PI material, which is adapted for the long-term working condition of the battery pack. In addition, the authors further highlight a highly practical demonstration of monitoring the battery surface pressure state by a film pressure sensor and placing the film sensor’s upper display interface on the driver’s console to achieve real-time monitoring of the on-board battery state. A higher accuracy instrument (laser displacement sensor) validated the designed sensor detection system (Fig. 7c). The results demonstrated that the flexible film pressure sensor was able to track the stress state of the battery very well and that it was highly compatible with the laser displacement sensor in the time domain. This advancement in detection technology is significant.

Notably, changes in surface pressure on the battery are attributable to variations in the pressure of the electrolyte. During the charging and discharging process, the battery undergoes periodic expansion and contraction (Fig. 7d), and the solid electrolyte interface (SEI) phase layer on the negative side of the battery grows and dissolves accordingly [133], which is a healthy state. With the elongation of the usage time, the battery gradually ages, generating dead lithium, and exhibiting abnormal thickening of the SEI, which may lead to the reduction of the battery capacity, the abnormal expansion of the battery, and even cause the deflagration. Monitoring these degradation effects (e.g., SEI growth) through external or implanted sensors is important for preventing danger and protecting life and property.

Inspired by tomographic imaging techniques [134], there is a great deal of interest in analyzing the internal operando of batteries using film sensors. Among other things, monitoring real-world battery degradation is crucial for a wide range of battery applications in different scenarios. Obtaining quantitative degradation information in manipulating commercial batteries is susceptible to the limitations of the type of signal being detected. Ravi S. Prasher et al. proposed a non-embedded detection and quantitative assessment scheme based on an attached thermal wave sensor by exploiting the strong dependence of the *k*_eff_ on the structural changes of the battery, using the effective thermal conductivity of the battery (*k*_eff_) as a quantitative indicator of battery degradation [97]. Figure 7e reveals the internal structure of a commercial battery containing a copper collector, anode, diaphragm, cathode, and aluminum collector. It also presents a thermal contact model that reveals how heat is transferred inside the battery and the mode of operation of the contact thermistor. Heat transfer within the battery works through a combination of multilayer structures and interfacial contact thermal resistance, where changes in battery thermal conductivity (*k*_eff_) directly reflect degradation mechanisms such as electrolyte consumption and lithium deposition. Attaching a non-embedded thermal wave sensor to the battery surface (Fig. 7f), in conjunction with thermal contact modeling, allows for noninvasive quantification of the sources of battery degradation (e.g., lithium coverage $\phi_{{{\mathrm{Li}}}}$). Figure 7g illustrates the relationship between *k*_eff_ and electrolyte thermal conductivity (k_*f*_) and lithium coverage ($\phi_{{{\mathrm{Li}}}}$), showing that as lithium coverage increases, thermal conductivity increases accordingly. The method is innovative in that it directly correlates the complex heat transfer process with the battery structural degradation, providing a new tool for the health management of commercial batteries.

### Battery Temperature Detection

**Fig. 8:**
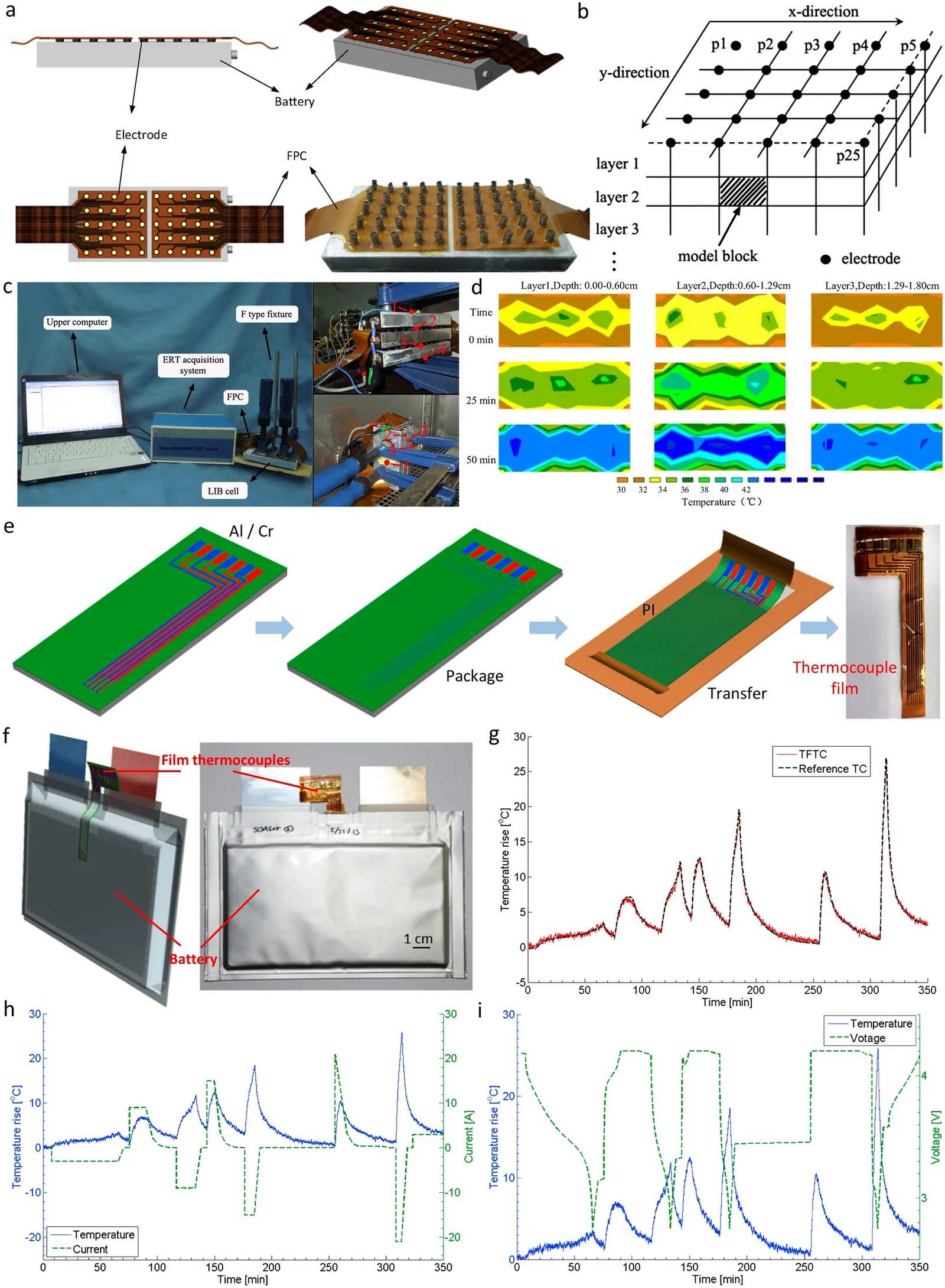
**a** Temperature–sensitive resistive tomography for monitoring the local temperature of an aluminum-cased lithium-ion battery. Copyright 2016, Elsevier. Reproduced with permission [83]. **b** Specific layout of the temperature–sensitive sensor model. Copyright 2016, Elsevier. Reproduced with permission [83]. **c** Test and acquisition system for monitoring by temperature–sensitive resistive tomography, and **d** Presentation of results. Copyright 2016, Elsevier. Reproduced with permission [83]. Copyright 2016, Elsevier. Reproduced with permission [83]. **e** Preparation process of film thermocouple sensors and f Working scenarios. Copyright 2014, Elsevier. Reproduced with permission [99]. Copyright 2014, Elsevier. Reproduced with permission [99]. **g** Thermocouple sensors temperature characterization during battery charging and discharging. Copyright 2014, Elsevier. Reproduced with permission [99]. **h** Current–temperature relationship and **i** Voltage–temperature relationship. Copyright 2014, Elsevier. Reproduced with permission [99]. Copyright 2014, Elsevier. Reproduced with permission [99]

Film temperature sensor detection technology is indispensable importance in battery health monitoring. Changes in battery temperature can have many effects on device performance, lifetime, safety, and reliability, so accurate temperature monitoring is critical. The unique advantages of film temperature sensors can be fully utilized and combined with the tomography technique to noninvasively obtain information on the internal temperature distribution of batteries. Michael Ho et al. proposed a method based on the electrical resistance tomography (ERT) technique to monitor the local temperature of aluminum-cased lithium-ion batteries. By designing a flexible printed circuit board sensor device conformally attached to the battery surface (Fig. 8a) [83], the relationship between apparent resistivity and local battery temperature and residual capacity was investigated. The residual capacity is used to evaluate the electrochemical reaction process inside the lithium-ion battery. Indeed, the state of charge (SOC) is described as the remaining percentage of the rated capacity and is widely used in the electronics industry. Figure 8b illustrates the specific layout of the model, which is divided into multiple layers and carries an array of sensors. The sensors are arranged in the x- and y-directions to monitor the temperature distribution inside the battery. They import the data to the upper computer (Fig. 8c) via the ERT acquisition system to realize the real-time monitoring of the internal temperature of the battery and verify its effectiveness in practical applications. Figure 8d shows the temperature distribution at different depths (Layer 1, Layer 2, and Layer 3) inside the battery for different time periods (0, 25, and 50 min). At 0 min, the temperature distribution is relatively even; at 25 min, the temperature begins to rise, and the color changes to more green; at 50 min, the temperature rises further and reaches a high value. During battery discharge, the internal temperature gradually increases over time. This temperature increase is related to the electrochemical reactions within the battery, specifically the movement of lithium ions between the positive and negative electrodes and the side reactions that occur in the electrolyte [135–137]. These reactions generate heat, leading to an increase in the internal temperature of the battery. The temperature distribution of the different depth layers shows the transfer and distribution of heat inside the battery, which is an exciting result. This technique provides a new solution for the thermal management of lithium-ion batteries.

Compared to film temperature sensors based on temperature effects, thermocouple sensors are more resistant to high temperatures and do not require an external excitation power supply, making them well suited to complex environments and avoiding electromagnetic interference caused by current excitation. Film thermocouple sensors play an essential role in battery health monitoring [138], especially temperature monitoring. In a study, Xiaochun Li et al. present a flexible thin-film thermocouple (TFTC) sensor technique for internal temperature monitoring of lithium-ion soft-pack batteries [99]. The technique enables in situ temperature monitoring by embedding a transferable, flexible film thermocouple in the battery (Fig. 8f) to improve battery safety and performance. TFTC is fabricated on a glass substrate and transferred to a copper foil, and a film of aluminum–nickel and Inconel alloy is deposited on the substrate by DC sputtering as a temperature sensing element, and polyimide is used as a flexible substrate (Fig. 8e), which is highly temperature resistant and chemically stable.

In actual measurements, the thermocouple sensor and the reference thermometer have a high degree of consistency in monitoring the battery temperature evolution as the battery is charged and discharged (Fig. 8g), which further demonstrates the excellent performance of the film thermocouple sensor. The temperature variation of the battery is caused by heat generation during charge/discharge cycles. The current–temperature and voltage–temperature relationship curves of the battery are plotted in Fig. 8h, i, respectively, to further reveal the heat generation characteristics of the battery during operation. During the discharge of the battery, its temperature shows an increasing trend, and the voltage shows a periodic decrease. The drop in voltage may be associated with an increase in the internal resistance of the battery, which likely results from due to the rise in temperature. The performance and lifetime of the battery may be negatively affected by the increase in internal temperature. Therefore, an effective thermal management system is required to control and mitigate the situation [92]. The combination of thermocouple sensor detection technology with a battery management system is expected to enable in situ monitoring of individual batteries.

### Multi-mode Sensing and Multiparameter Detection

**Fig. 9:**
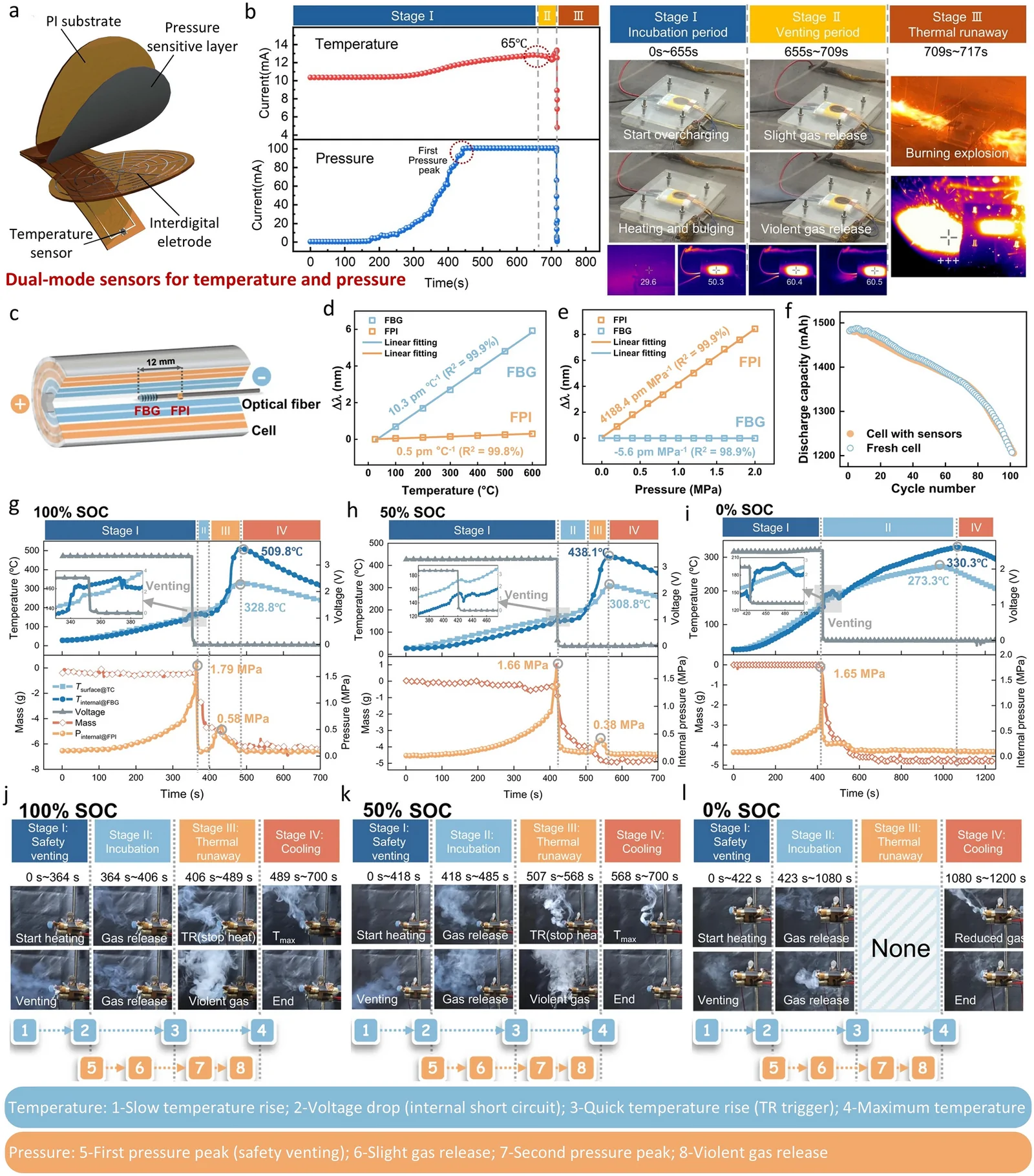
**a** Dual-mode sensors for temperature and pressure. Copyright 2025, Elsevier. Reproduced with permission [96]. **b** Dual-mode sensors for independent sensing and battery status tracking. Copyright 2025, Elsevier. Reproduced with permission [96]. **c** Schematic of the FBG sensor implanted inside the battery. Copyright 2023, Springer Nature. Reproduced with permission [78]. **d** Wavelength versus temperature for the FBG sensor in the range of 25–600 °C. Copyright 2023, Springer Nature. Reproduced with permission [78]. **e** Wavelength versus pressure for the FPI in the range of 0–2 MPa pressure. Copyright 2023, Springer Nature. Reproduced with permission [78]. **f** Impact of the implantation of the FBG–FPI sensor on the performance of the recycling of a commercial 18,650 Li-ion battery. Copyright 2023, Springer Nature. Reproduced with permission [78]. Characterization of battery thermal runaway at **g** 100% SOC, **h** 50% SOC and **i** 0% SOC. Copyright 2023, Springer Nature. Reproduced with permission [78]. Copyright 2023, Springer Nature. Reproduced with permission [78]. Copyright 2023, Springer Nature. Reproduced with permission [78]. Measured states of battery thermal runaway at **j** 100% SOC, **k** 50% SOC and **l** 0% SOC. Copyright 2023, Springer Nature. Reproduced with permission [78]. Copyright 2023, Springer Nature. Reproduced with permission [78]. Copyright 2023, Springer Nature. Reproduced with permission [78]

Temperature and pressure changes are critical warnings for health monitoring of lithium batteries. In addition to the pressure and temperature film sensor types described above, Minghua Chen et al. reported a dual-mode sensor for temperature and pressure (Fig. 9a) for real-time monitoring of lithium battery operando and thermal runaway [96]. The ability of the dual-mode sensor to simultaneously monitor both parameters at the same location was the most critical aspect of the development of this sensor. The two sensors can be sensed independently (Fig. 9b) and tracked throughout the battery’s life cycle. The thermal runaway of the battery is divided into three phases (incubation period, venting period, and thermal runaway), and the field images and thermal imaging photographs record the moments of these processes. It can be found that in the incubation period, the battery temperature and expansion force gradually increase. During the venting period, the temperature gradually destabilizes and the pressure rapidly fluctuates-a process lasting only 23 s. Subsequently, the battery undergoes thermal runaway, and the sensor is destroyed. The application of dual-mode sensors makes it possible to monitor the two battery parameters synchronously, making it one of the research trends for future development.

Additionally, cylindrical batteries are commercially prevalent [139, 140]. For these curved batteries, the method of attaching film sensors may have limitations. The powerful capability of FBG can be applied not only to the external electrodes of the battery but also implanted inside the battery. Tuan Guo et al. realized experiments in the application of FBG sensors to commercial 18,650 batteries for continuous monitoring of the internal temperature and pressure of the batteries [78], and have successfully observed a stable and reproducible optical response in lithium-ion batteries associated with thermal runaway. The way the FBG sensor is embedded inside the battery is shown in Fig. 9c. It can be found that the FBG sensor consists of two components, FBG and Fabry–Perot interferometer (FPI). FBG realizes the sensing of temperature and FPI realizes the sensing of pressure. They are both analyzed by spectral variations, and both their resonance wavelengths exhibit highly linear relationships to temperature (Fig. 9d) and pressure (Fig. 9e). The sensitivity of the FBG to temperature is 10.3 pm °C^−1^, and the sensitivity to pressure is only − 5.6 pm MPa^−1^. While the sensitivity of the FPI to pressure is 4,188.4 pm MPa^−1^, and the sensitivity to temperature is 0.5 pm °C^−1^. The organic combination of FBG and FPI allows the sensor to fully sense both temperature and pressure and achieve decoupling (essentially ignoring the FBG response to pressure and the FPI response to temperature). The FBG–FPI sensor was implanted into a commercial 18,650 Li-ion battery, and the effect of sensor implantation on battery performance was evaluated. Figure 9f shows that the battery implanted with the FBG–FPI sensor exhibits comparable performance over 100 charge/discharge cycles compared to the battery without the sensor implant. This demonstrates that the sensor implantation has a very limited impact on the battery performance, providing the possibility of fiber optic sensors for operando testing of commercial batteries.

In practical applications, three SOCs, 100%, 50%, and 0% SOC, are selected to analyze the thermal runaway of the battery. Figure 9g–i shows the real-time behavior of the internal temperature, pressure, mass loss, and output voltage of the battery during thermal runaway. It is divided into four phases. After the heater is switched on, the battery surface temperature rises rapidly as the safe ventilation stage (Stage I); the electrolyte releases vapor gas, causing mass loss and a sudden drop in pressure as the latency stage (Stage II); the white jet of smoke and the temperature continue to rise to a maximum of 509.8 °C (or 438.1 °C, 330.3 °C) in the thermal runaway stage (Stage III); the end of the reaction with no mass loss and gas production and the temperature gradually returning to the ambient temperature is the cooling stage (Stage IV). The measured states are shown in Fig. 9j–l. Research on commercial 18,650 lithium batteries has shown that fiber optic implantation inside the batteries can monitor key parameters such as temperature, pressure, refractive index, gas, and SEI growth [141]. This is critical for operational safety, enabling early detection of hazardous situations such as thermal runaway, overpressure, and side reactions. It is also valuable for battery health management, providing insight into reaction processes, predicting lifetime, and optimizing charge and discharge strategies to improve performance and utilization and reduce operating costs.

**Fig. 10:**
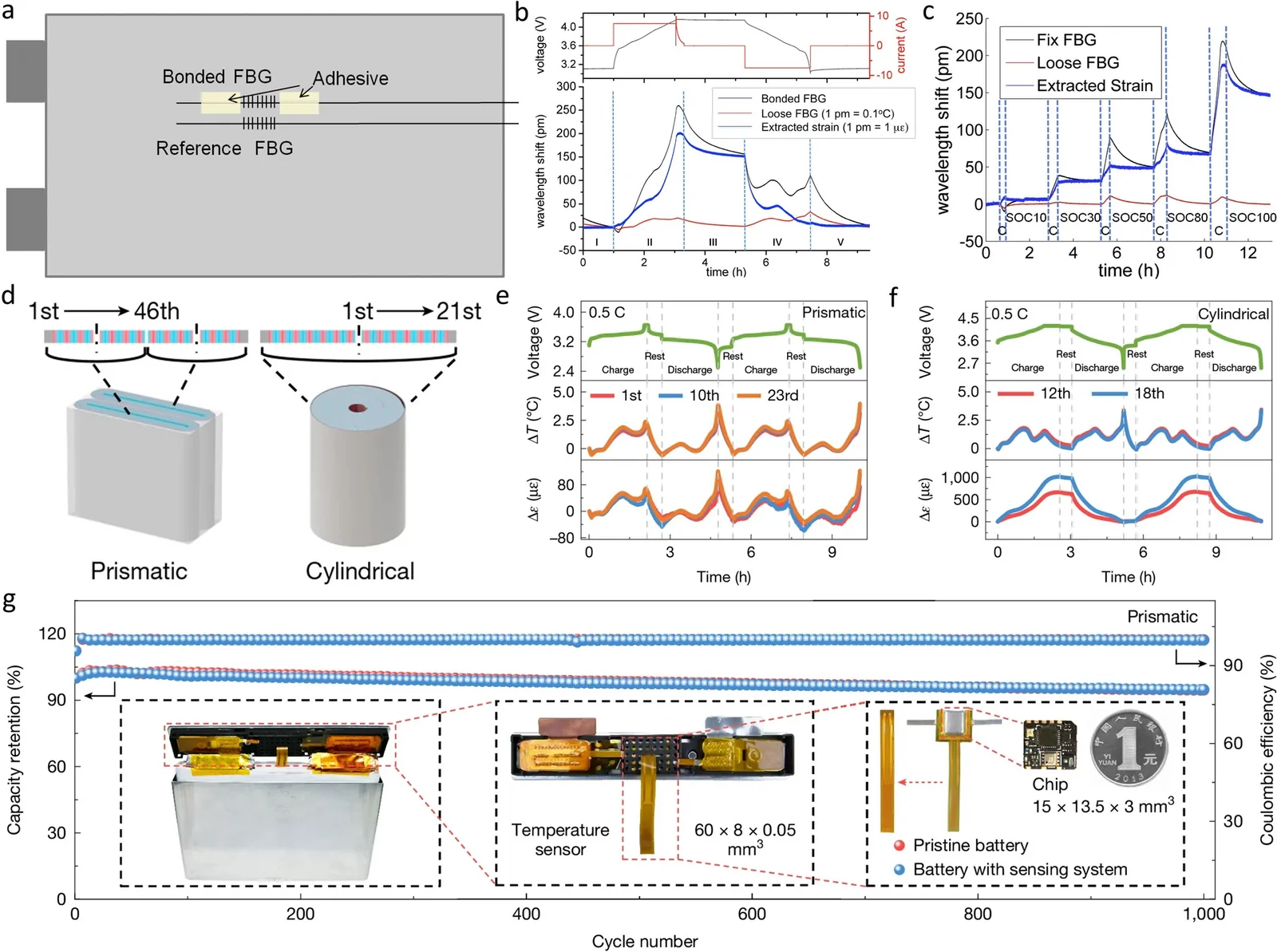
**a** FBG sensor applied to a pouch battery. Copyright 2015, Elsevier. Reproduced with permission [102]. **b** Wavelength offsets over time measured by the combined FBG sensor, the loosely attached reference FBG sensor, and the temperature-compensated strain signals. Copyright 2015, Elsevier. Reproduced with permission [102]. **c** Wavelength offsets of the combined FBG sensor and the reference FBG sensor, and the extracted strain signals. Copyright 2015, Elsevier. Reproduced with permission [102]. **d** Applications of sensors in prismatic and cylindrical batteries. Copyright 2025, Springer Nature. Reproduced with permission [142]. **e** The voltage, internal temperature, and strain profiles of prismatic and f cylindrical batteries during operation at 0.5 C are shown. Copyright 2025, Springer Nature. Reproduced with permission [142]. Copyright 2025, Springer Nature. Reproduced with permission [142]. **g** The capacity retention and coulombic efficiency of battery at 0.5 C over 1000 cycles. Copyright 2025, Springer Nature. Reproduced with permission [142]

In a study, Ajay Raghavan et al. revealed a complex coupling mechanism between volumetric strain relaxation and state of charge (SOC), temperature during battery charging, and discharging by innovatively deploying an FBG sensing system on the surface of a soft-packed lithium-ion battery (Fig. 10a) [102]. Figure 10b illustrates the wavelength shift of the combined FBG sensor, the loosely attached reference FBG sensor, and the temperature-compensated strain signal measurements over time during a standard cycle. In this case, the reference sensor is only sensitive to temperature changes at the battery surface. The standard cycle is divided into five regions: the initial resting period (I), the standard charging period (II), the resting period after charging (III), the standard discharging period (IV), and the resting period after discharging (V). It can be noticed that the reflected wavelengths of both sensors are zeroed at the beginning of the charging period. A wavelength shift of 1 pm corresponds to a strain of about 1 με in the extracted strain signal (or a temperature change of about 0.1 °C in the loose FBG signal). The wavelength shift of the reference sensor increases predominantly during charging and discharging, which is due to the temperature increase caused by the increase in internal resistance (e.g., polarization resistance and associated Joule heating). However, there are also different SOC values during charging and discharging, during which a heat absorption response can be observed. The relationship between strain relaxation and SOC was examined by gradually charging to different SOC levels. The battery was gradually charged to 10%, 30%, 50%, 80%, and 100% SOC levels at a rate of C/2 and rested for 2 h at each SOC level. Figure 10c shows the wavelength offsets of the combined FBG sensor and the reference FBG sensor, as well as the extracted strain signals. The wavelength shift of the reference FBG reflects the temperature change during each charging step, as the temperature gradually returns to the initial temperature during the subsequent rest phase. During the rest phase after charging, the strain signals show different characteristics at different SOCs: at low SOCs, there is no significant relaxation, while at high SOCs, a significant relaxation process is observed. In particular, the relationship between strain relaxation and SOC (total amount of embedded/de-embedded lithium) will be directly related to the stability and safety of the battery.

It is noteworthy that the strain evolution of prismatic and cylindrical batteries differs significantly. Wei-Li Song et al. detected temperature and strain signals from jelly roll ribbons in both prismatic and cylindrical batteries (Fig. 10d) [142]. For both battery types, temperature varies with SOC and reaches its maximum at the end of discharge (Fig. 10e, f). This temperature–SOC relationship is highly consistent. However, strain evolution differs markedly: strain in prismatic cells is temperature-dependent, mirroring temperature evolution. In contrast, strain evolution in cylindrical cells exhibits significant layer-to-layer variation, approximately 350 με. This disparity may stem from graphite phase transitions [143]. In summary, strain evolution exhibits high correlation with the temporal distribution of SOC, changing in tandem with SOC depletion. Figure 10g demonstrates that the battery retains over 90% capacity after 1000 charge–discharge cycles, indicating slow SOH degradation. Even with sensors implanted, the battery maintains exceptionally high performance.

To emphasize, integrating multiple sensor types into a unified fusion framework can effectively mitigate spatiotemporal aliasing effects. In large-scale battery packs, inconsistencies in sampling and response times between stress, strain, and temperature sensors can trigger aliasing, leading to blurred or misaligned critical events across different modalities. Multi-channel signal synchronization and missing time window interpolation techniques achieve multi-sensor spatiotemporal synchronization, enabling high-fidelity reconstruction of coupled mechanical–thermal–electrochemical behavior.

To enhance interpretability, AI models were developed to separate overlapping mechanical and thermal signals within the same sensor channel. For instance, in dual-mode temperature–pressure sensors, pressure-induced resistive changes in film sensors often coexist with temperature-induced resistive changes, complicating feature attribution. By applying time–frequency decomposition (such as wavelet transform) and feature engineering (such as separating low-frequency thermal drift from high-frequency mechanical oscillations), machine learning models extract orthogonal feature sets. Furthermore, training processes constrained by physical principles (such as enforcing thermodynamic monotonicity for temperature features and elastic linearity for stress features) effectively decouple mechanical and thermal effects, thereby enhancing model interpretability.

## AI-Enabled Data Processing

**Fig. 11:**
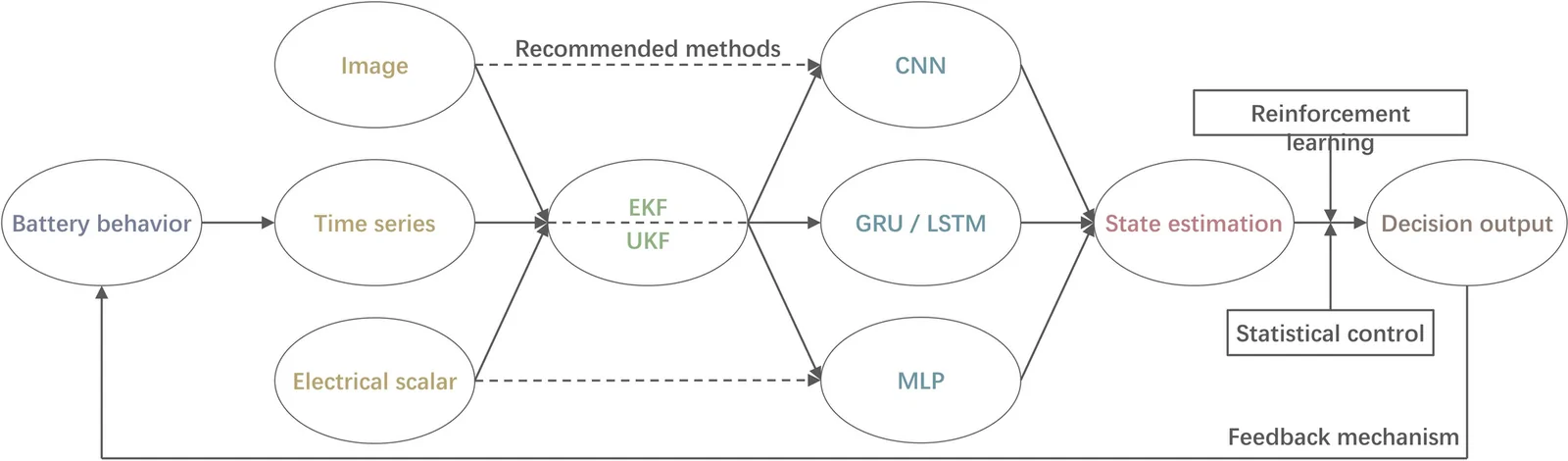
AI-enabled data processing

Flexible sensing technology provides a breakthrough solution for real-time monitoring of multi-physical field states under complex battery operando. However, the massive multidimensional data generated by these sensors presents challenges for interpretation and timely decision-making. To address this, AI plays a pivotal role in data fusion and predictive analysis. By integrating flexible sensing with AI algorithms, a closed-loop “sensing–analysis–control” framework can be constructed, which substantially enhances monitoring accuracy and system reliability. Taking film pressure sensor as an example, it starts from the original signal generated by battery behavior. The strain–temperature signal output by the sensor undergoes preprocessing via an extended/unscented Kalman filter (EKF/UKF) to eliminate environmental noise and complete data calibration. Subsequently, feature points related to battery behavior are extracted within the algorithmic model. A common approach is a modular fusion architecture, which serves as a universal model. A convolutional neural networks (CNN) encoder extracts spatial temperature features, while a gated recurrent units or long short-term memory (LSTM) encoder models the strain time series. A multilayer perceptron (MLP) encoder processes the electrical signal. Subsequently, the state estimation module couples the battery voltage relaxation curve with temperature field distribution data. Finally, the decision output generates health status classification alerts based on dynamically determined thresholds, driving the battery management system. Notably, dynamic threshold determination typically employs statistical control or reinforcement learning approaches to guide decision output. Statistical control employs window-based mean, variance, and uncertainty estimation to manage false alarm rates, whereas reinforcement learning models alarm decisions as a Markov decision process, utilizing policy learning for adaptive thresholding. Statistical control offers low computational complexity and high sampling rates, while reinforcement learning delivers synergistic effects, enabling threshold determination and coordinated control decision-making under complex, multimodal operating conditions (Fig. 11). Such a sensing-AI collaborative mechanism elevates battery health monitoring from passive response to active prevention.

In the context of large-scale application of lithium-ion batteries in electric vehicles [144], energy storage power stations, and other key areas, accurate monitoring of battery health status and remaining service life has become the core challenge to ensure system safety and economic benefits. However, limitation of traditional battery management systems lies in their reliance on shallow data analysis driven by artificial experience: in the face of the massive sensing data generated by the coupling of multiple physical fields during battery aging, the system is only able to extract limited dimensional explicit features (e.g., capacity degradation, internal resistance increase), but it is difficult to capture implicit degradation features, such as microwave motions in the nonlinear curve of the voltage relaxation and the impedance spectral phase angle shift. The rapid development of AI technology has significantly enhanced to battery health detection systems [145], which can process and analyze huge amounts of data (deep learning and machine learning algorithms, etc.) to improve the accuracy of monitoring and can also monitor key parameters in real-time according to the battery health status [146]. The system can formulate optimized charging strategies based on usage habits and battery status to improve charging efficiency and reduce energy consumption and can also significantly improve the time to warning and increase the operational efficiency and safety of the energy storage system.

**Fig. 12:**
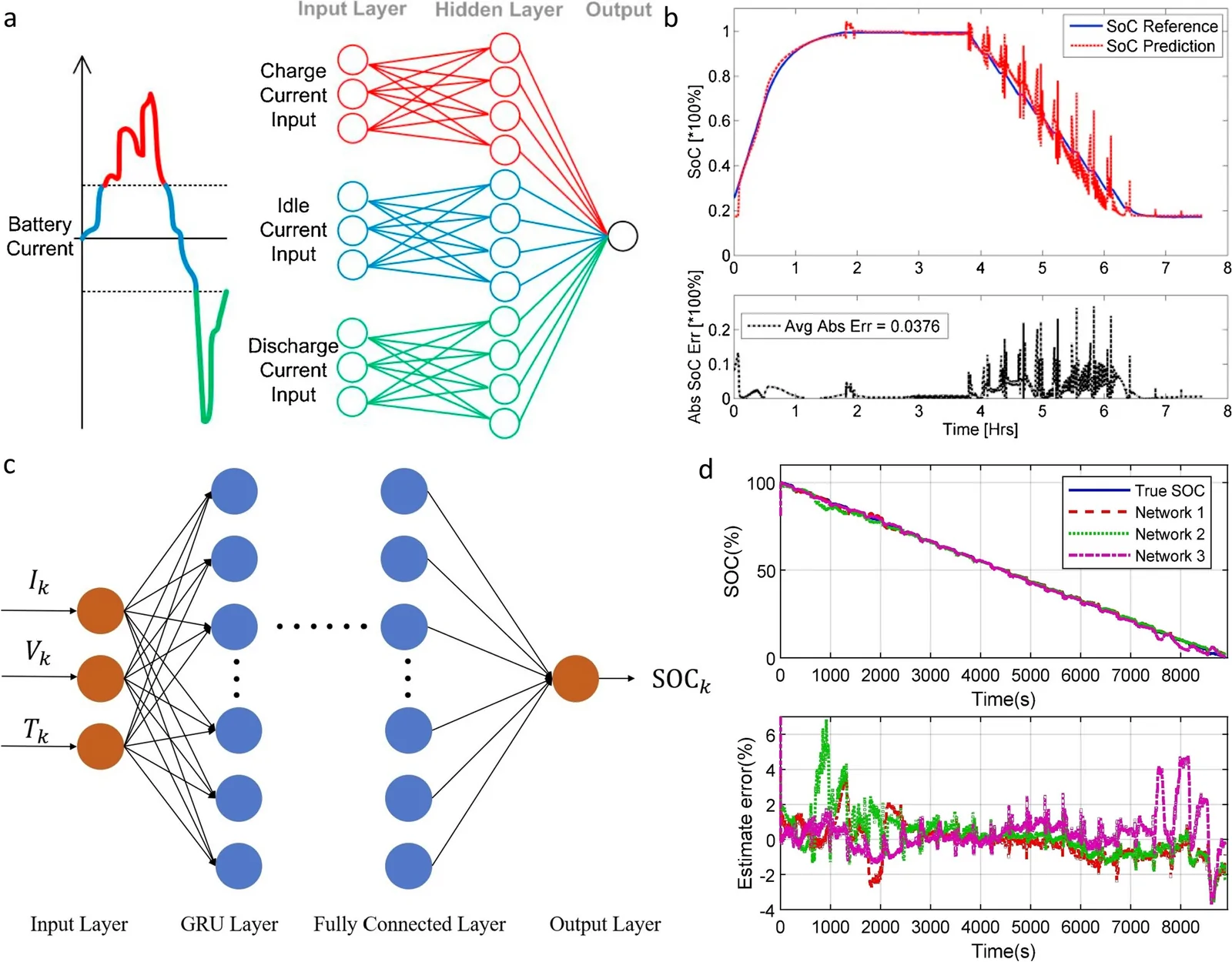
**a** Load classification neural network for battery SOC estimation. Copyright 2016, Elsevier. Reproduced with permission [147]. **b** Characterization of reference, prediction, and estimation error of SOC values. Copyright 2016, Elsevier. Reproduced with permission [147]. **c** GRU-RNN for SOC estimation of lithium-ion batteries. Copyright 2019, Elsevier. Reproduced with permission [148]. **d** When tracking battery SOC, the GRU-RNN model produces estimated values are in high agreement with the actual values. Copyright 2019, Elsevier. Reproduced with permission [148]

In a study, Jae Wan Park et al. proposed a method for battery SOC estimation using load classifying neural networks [147]. The method was developed by classifying the battery operating modes into resting, charging, and discharging scenarios and training a separate neural network model for each mode (Fig. 12a). The model was trained through vehicle driving cycle load profiles and validated using pulse test cycles, which showed an average estimation error of 3.8% (Fig. 12b). When the pulse test cycle undergoes a rapid load change, the magnitude of the error peaks decreases, but the number increases, and their predictions, augmented by the neural network, are also closer to the reference value. In another study, Qiang Miao et al. proposed a GRU-based RNN for SOC estimation of Li-ion batteries (Fig. 12c) [148]. The method trains the network by using current, voltage, and temperature signals, and compared to the load classification neural network, the recurrent neural network improves the SOC estimation accuracy by capturing the history better and performing repeated iterations. The problem of gradient vanishing in traditional RNNs is solved by GRUs, which are able to better capture long-term dependencies. GRUs improve the efficiency of utilizing historical information by deciding which information should be forgotten or retained through reset gates and update gates. Figure 12d shows the GRU-RNN tracking battery SOCs, and it can be found that the estimated values are in high agreement with the actual values with very small error values. The method achieves accurate estimation under dynamic loads and is robust to unknown initial SOC values and ambient temperature variations.

**Fig. 13:**
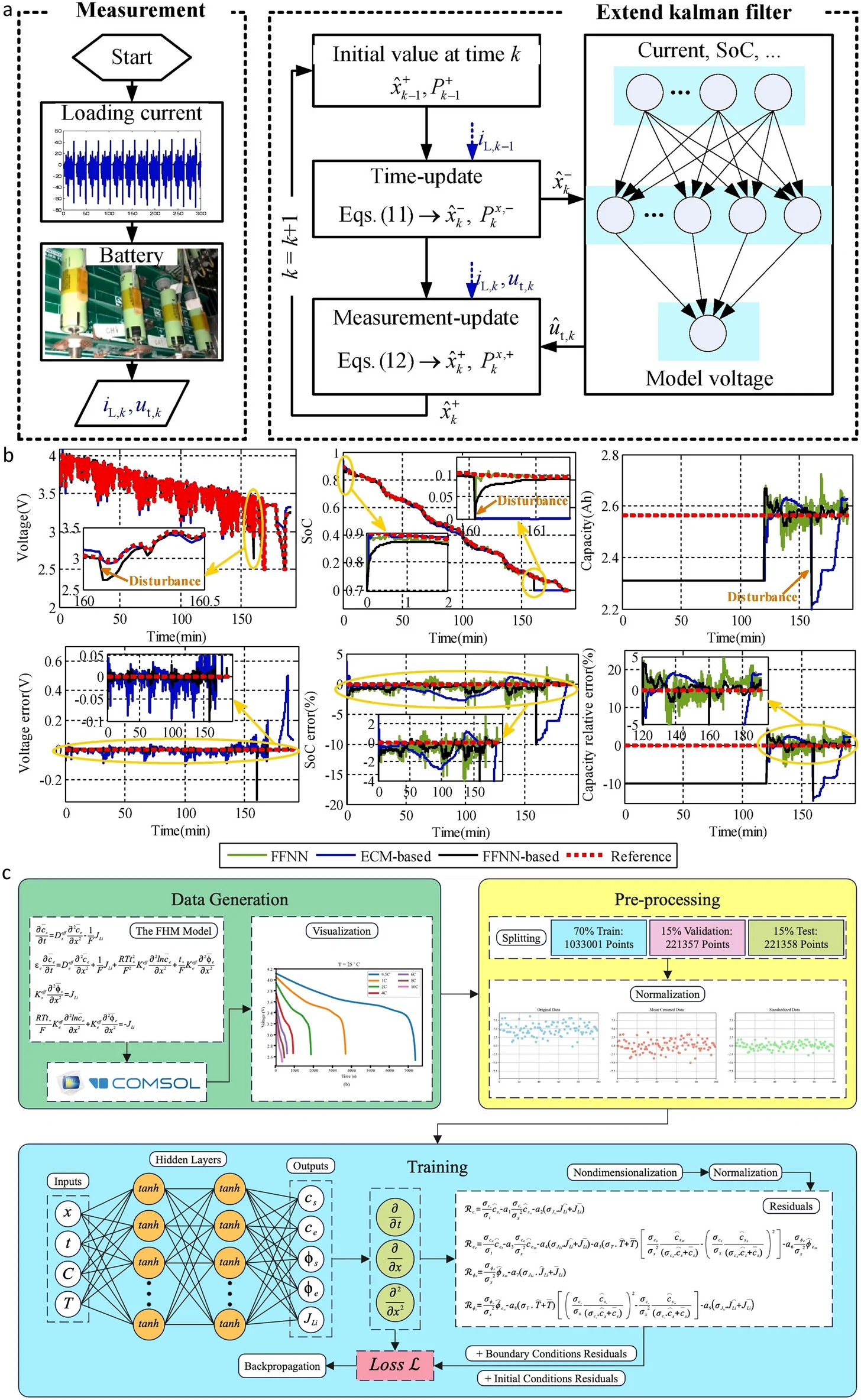
**a** Workflow of data measurement, KEF filtering, and SOC estimation in a battery management system. Copyright 2019, Elsevier. Reproduced with permission [149]. **b** Performance estimation with unknown initial battery capacity. Copyright 2019, Elsevier. Reproduced with permission [149]. **c** A framework for predicting battery behavior using the PINN model. Copyright 2025, Elsevier. Reproduced with permission [150]

The strong nonlinear characteristics of the battery system (e.g., the battery’s own nonlinearity, charging/discharging hysteresis effect, and complexity of the application environment) will lead to a complex dynamic coupling between the raw signal of the sensor and the real physical state, particularly at low temperatures, high multiplicity, or deep cycling and other operando, which will cause significant distortion of the voltage/current signal. Consequently, an enhanced feed-forward neural network (FFNN) and an EKF have been proposed by Fengchun Sun et al. for SOC estimation in lithium-ion batteries. As illustrated in Fig. 13a, the workflow of data processing and SOC estimation in a battery management system consists of two primary components: measurement and EKF [149]. First, in the measurement part, the system starts to load the current and obtains the current and voltage data through the battery. Subsequently, in the EKF part, the system uses the initial values for time update and measurement update. The neural network then performs the prediction and correction. This enables real-time monitoring and optimization of battery performance and state. In practice, Fig. 13b shows the performance estimation of the algorithm with unknown initial battery capacity, including the estimated and measured voltage value, SOC value, and capacity and their corresponding errors. It can be seen that even if there is a deviation in the initial setting of the capacity, the SOC estimation method using the FFNN and EKF-based SOC estimation method is still able to quickly and accurately converge to the true value, and its estimation error is always kept within 2%. This indicates that the algorithm is robust and efficient and can provide reliable SOC estimation in practical applications and maintain high accuracy even in the face of uncertainties.

Notably, lithium batteries exhibit segregation and delayed electrolyte ion diffusion at low temperatures, leading to discrepancies between predicted and actual SOC values. Model prediction methods based on GRU and EKF primarily rely on data-driven dynamics or simplified equivalent circuit assumptions, rendering them insensitive to data deviations caused by the battery ‘s nonlinear physical characteristics. Therefore, Ayat Gharehghani et al. proposed embedding a physics-informed neural network (PINN) into a fully homogeneous macro (FHM) model to predict key electrochemical parameters under varying loads and temperatures (Fig. 13c) [150]. This framework directly embeds physical models into the neural network’s predictive architecture, enabling it to adhere to physical constraints while leveraging data-driven adaptability. This enhances extrapolation capabilities under extreme conditions. By incorporating physics- and electrochemistry-based constraints, the framework reduces reliance on extensive experimental data and ensures physically consistent estimates. The PINN-enhanced SOC estimation framework significantly improves AI’s ability to construct battery management systems under extreme operating conditions.

AI-enabled battery health monitoring significantly improves battery safety and reliability by analyzing and predicting the state of lithium-ion batteries in real time [151]. By accurately estimating the battery’s SOC and SOH, AI is able to predict potential thermal runaway or other malfunctions so that measures can be taken in advance to avoid safety incidents. In addition, AI can optimize battery use and maintenance strategies to extend battery life and reduce replacement frequency and operating costs [152].

**Fig. 14:**
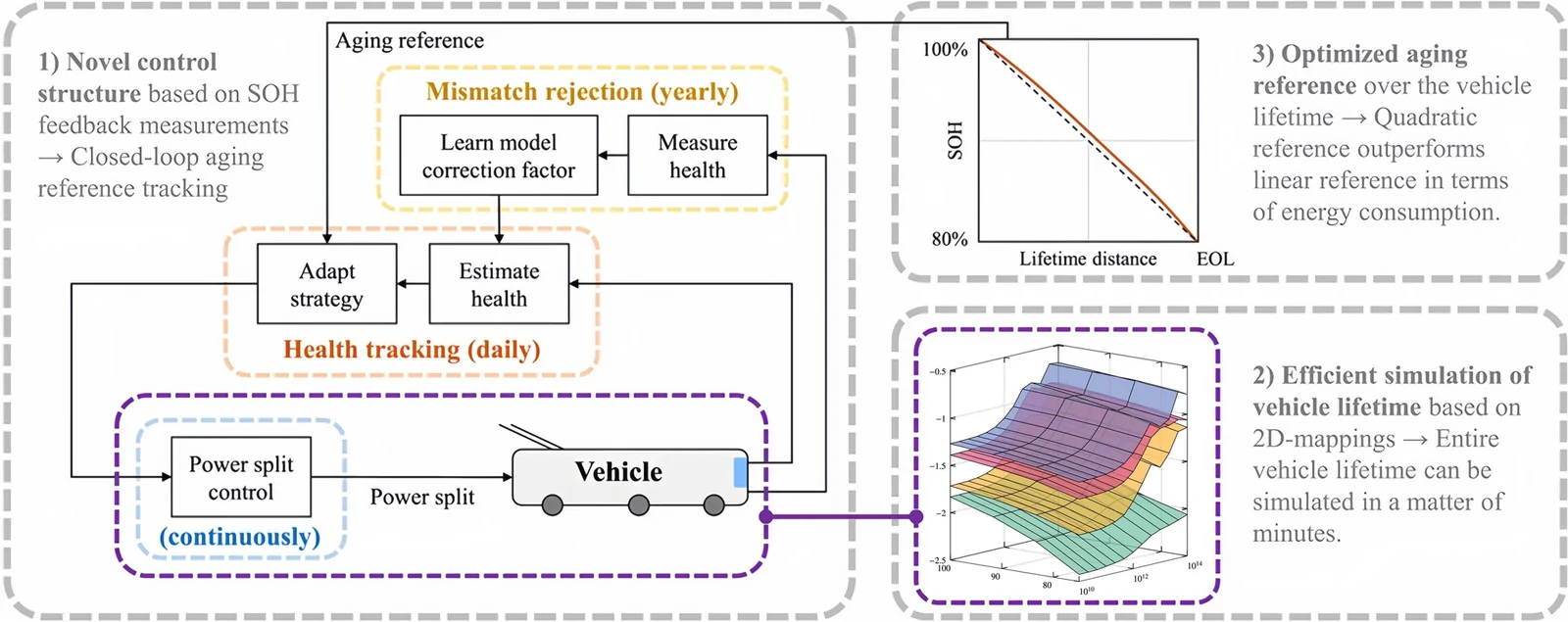
Closed-loop control system for battery health status. Copyright 2023, Elsevier. Reproduced with permission [153]

Although the deep integration of flexible sensing technology and AI provides a revolutionary tool for battery health monitoring, its synergistic application still faces multiple challenges. 1) Multi-data fusion: multiple types of data need to be strictly on their time scales to facilitate data decoupling; 2) Model generalization capability: Existing AI models (e.g., GRU, EKF) can have an error of less than 2% when trained in a laboratory environment, but under actual working conditions, due to temperature disturbances, mechanical vibrations, and other noise influences, the error may increase to more than 10%; 3) Closed-loop control of the system: From the acquisition of sensing data to the execution of regulation commands needs to be completed within 100 ms, which puts strict requirements on the hardware operation. In response to the above challenges, mainstream AI techniques present differentiated advantages: GRU has efficient time-domain modeling capability; EKF has strong robustness and is difficult to cope with model drift; PINN can achieve a high level of training prediction with fewer samples, but their complexity is higher. In the practice of closed-loop control, it is necessary to combine the “sensing-AI” synergy strategy with the digital twin platform to optimize the training ability and improve the generalization ability so as to provide support for the scale-up of high-security energy storage systems. Fabio Widmer et al. proposed a closed-loop control system and an efficient simulation method for battery health state-oriented life prediction and health state assessment of on-board batteries (Fig. 14) [153]. The lifetime simulation of batteries is achieved by abstracting complex dynamic behaviors into 2D mappings, followed by fast computation through interpolation. This excellent simulation mechanism allows years-long lifetime simulations to be quickly completed in minutes, greatly improving efficiency. Reacting to the health state through the secondary reference trajectory of the battery, which is superior to the linear reference, forms a feedback to the health monitoring and realizes the accurate tracking of the battery health state, providing an innovative solution for the battery management of electric vehicles.

Recent studies have demonstrated the potential of AI-enabled models for SOC and SOH estimation with quantitative benchmarks. To provide a more quantitative comparison of AI-enabled strategies for battery state estimation, representative studies from the recent studies are summarized in Table 3. The results highlight how different combinations of feature inputs, preprocessing procedures, and model architectures translate into distinct levels of performance. Specifically, simple NN classifiers applied to voltage and current achieve mean squared errors below 3.8% [147], while the GRU–RNN model further reduces SOC estimation errors to < 3.5% RMSE under dynamic conditions [148]. Hybrid approaches combining FFNN with EKF filters can achieve high convergence speed and SOC errors < 2% [149], whereas PINN incorporates electrochemical constraints to enhance generalization with RMSE = 3.89% and MAE < 2% [150]. Beyond SOC estimation, closed-loop control frameworks integrating rapid kinetic mapping and simulation demonstrate accurate SOH estimation with mean absolute deviations as low as 0.9% [153].

**Table 3:** Quantitative analysis of the AI-enabled process

Feature input	Preprocessing	Model class	Task	Quantitative results	References
Voltage, current	Load profile classification, normalization processing	NN	SOC estimation	Mean squared error (MSE) < 3.8%	[147]
Voltage, current, temperature, SOC	Normalization processing, data augmentation	GRU, RNN	SOC estimation	Root mean square error (RMSE) < 3.5%, mean absolute error (MAE) < 2%	[148]
Voltage, current, temperature, SOC, polarization state	Polarization state calculation, normalization processing, time constant selection	FFNN, EKF	SOC estimation	RMSE = 0.05 V, SOC estimation error < 2%	[149]
Voltage, current, temperature, SOC, electrolyte concentration, electrode potential	Normalization processing, dimensionalization, dataset partitioning	PINN	SOC estimation	RMSE = 3.89%, MAE < 0.02 V, MAE < 2%	[150]
SOC, SOH, aging characteristics	OpenSesame model, rapid kinetic mapping	Closed-loop control, simulation	SOH estimation	Mean absolute deviation (MAD) = 0.9%	[153]

AI-enabled battery health monitoring technology promotes the intelligent process of battery management. Through the coupling of flexible sensing with algorithms such as deep learning and dynamic filtering, it parses multidimensional data, senses key features inside the battery, and improves the accuracy and timeliness of health status assessment. The introduction of the closed-loop control system further amplifies the unique advantages of AI empowerment, using the powerful arithmetic power of AI to improve the accuracy of the assessment, while combining real-time feedback and system simulation. Additionally, AI-enhanced closed-loop control significantly enhances battery pack safety. By integrating state variables derived from sensors with predictive models, the system can generate anomaly alerts and implement corrective actions within 100 ms. This capability demonstrates that closed-loop integration not only accelerates the response time of battery management systems but also directly improves reliability and safety margins at both the module and battery pack levels.

## Summary and Outlook

**Fig. 15:**
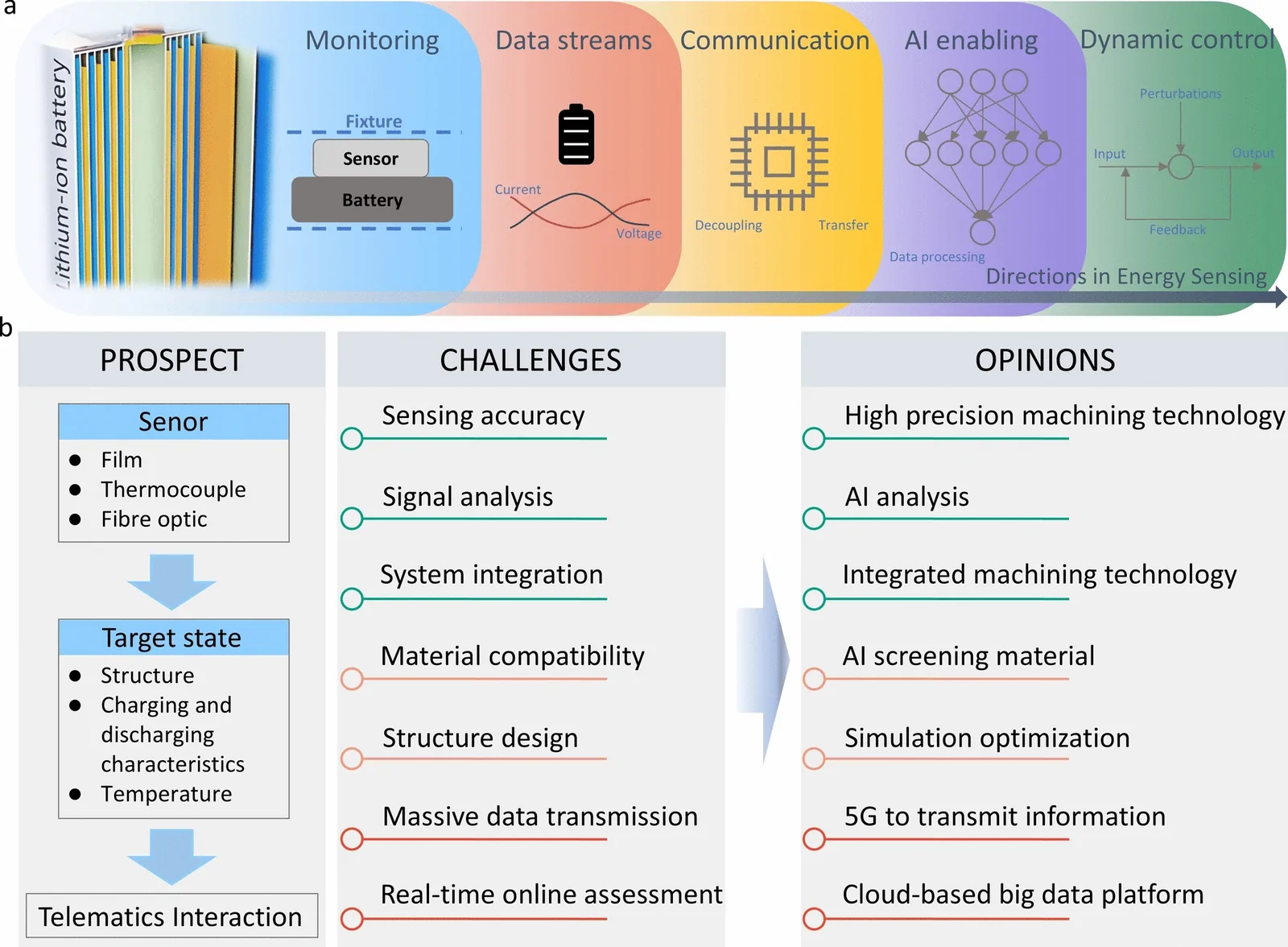
**a** Directions in energy sensing; **b** Current scientific issues facing flexible sensors in battery health monitoring and technical pathways to address these challenges

This paper reviews the research progress of flexible sensors for battery health monitoring. Flexible sensors have important application prospects in battery health monitoring, enabling real-time monitoring of the battery state, including key parameters such as structure, charging and discharging characteristics, and temperature. By using different types of sensors (e.g., film sensors, thermocouples, and fiber optic sensors), multidimensional monitoring of batteries can be achieved. Currently, numerous studies are based on analyzing the state of the battery through pressure control and battery charging and discharging systems, followed by decoupling and communication of the data. In this process, the sensors will generate a substantial number of multidimensional data streams, in which a multitude of parameters pertaining to the battery status will be obscured. The emergence of AI technology can well assist in data feature extraction and analysis and timely feedback to the user and monitoring system. This will be the future direction of battery energy sensing (Fig. 15a). Through the synergistic operation of the five core modules monitoring, data flow, communication, AI enabled, and dynamic control, a closed-loop system from real-time collection of battery status (e.g., parameters such as current, voltage, capacity, etc.) by sensors to intelligent analysis of data, and then optimization of the battery performance through dynamic feedback is realized. Among them, AI technology runs through data decoding, feature extraction, and decision control, integrating with decoupled transmission and an instant feedback mechanism, which not only improves the accuracy and response speed of battery status monitoring but also actively regulates the charging and discharging process through intelligent algorithms, which provides a visual solution for battery safety early warning, life prediction, and energy efficiency optimization.

### Future Challenges and Development Paths

Although flexible sensing technology combined with artificial intelligence shows tremendous potential in advancing battery health monitoring, its large-scale deployment still faces significant challenges [154, 155], which may translate into opportunities for sustainable development. Current flexible sensors used for battery health monitoring exhibit shortcomings in several critical areas, including sensing accuracy, signal analysis, system integration, material compatibility, structural design, massive data transmission, and real-time online assessment (Fig. 15b). Multiple technical pathways can alleviate these bottlenecks: employing high-precision machining technologies to enhance sensor fabrication accuracy and consistency [156]; utilizing AI for data analysis to improve processing efficiency and accuracy; developing integrated machining technologies that combine multiple sensors with data processing modules into a single system to ensure stability and reliability; optimizing sensor material selection through intelligent screening techniques to boost performance and reliability [157]; employing simulation techniques to optimize sensor structural design for adaptability to diverse battery configurations and environmental applications; adopting 5G technology to enable massive data transmission and ensure transmission efficiency; establishing cloud-based big data platforms for centralized data management, and developing real-time online evaluation technologies to deliver more comprehensive battery health monitoring services.

Looking ahead, the development of flexible sensing technology needs to closely focus on the three main lines of “long-term reliability,” “system integration and packaging,” and “data closed-loop control.” At the material level, explore self-healing and corrosion-resistant materials to extend sensor service life in extreme environments. At the system integration level, explore wireless passive sensor networks transmission and communication technologies to address challenges in high-density deployment of large-scale battery modules. At the data level, leverage large-scale sensor deployment to increase training data for AI models under operando conditions, and enhance the scientific accuracy of condition prediction through digital twin technology. It is foreseeable that flexible sensing technology will deeply reshape the pattern of battery health monitoring, from the multiparameter sensing of the battery monomer to the collaborative management of the module and then to the closed-loop feedback digital twin platform, which will greatly improve the safety threshold of the battery.

### Computational Challenges and Model Generalization

Despite significant advancements in AI-enabled battery health monitoring technologies, several challenges remain before achieving large-scale deployment:

1. *Error propagation under actual operating conditions* SOC estimation models based on GRU and EKF achieve < 2% estimation error in laboratory environments, but their robustness significantly degrades under real-world operating conditions. For instance, in mechanically vibrating environments, noise coupling between strain and voltage signals may infiltrate the estimation loop, causing SOC deviations exceeding 10%.
2. *Data requirements and annotation costs* High-performance deep learning models typically require training on multimodal sensor data (including stress, strain, temperature, and voltage/current) spanning 10^4^–10^5^ cycles. Constructing such comprehensive datasets necessitates extensive cyclic testing and precise real-value annotation (e.g., capacity decay), resulting in substantial costs.
3. *Hardware constraints* In practical battery management systems, closed-loop decision-making from sensing to execution must occur within < 100 ms to enable timely thermal runaway warnings. This time constraint limits computational complexity, posing unique challenges for large-scale neural networks.
4. *Model generalization* Existing AI models exhibit limited generalization when transferred from laboratory environments to diverse actual scenarios. PINN with electrochemical constraints into learning frameworks enhances extrapolation capabilities but increases computational complexity.

To overcome these computational bottlenecks, collaboration is needed across three key domains: 1) developing physically constrained AI architectures to suppress error propagation; 2) establishing standardized large-scale open datasets to reduce redundant annotation costs; 3) implementing hardware-algorithm co-design to ensure response times below 100 ms. These directions are critical for bridging the gap between proof-of-concept and industrial deployment of AI-based flexible sensing systems.
